# Lipidomics reveals the reshaping of the mitochondrial phospholipid profile in cells lacking OPA1 and mitofusins

**DOI:** 10.1016/j.jlr.2024.100563

**Published:** 2024-05-18

**Authors:** Andrea Castellaneta, Ilario Losito, Vito Porcelli, Serena Barile, Alessandra Maresca, Valentina Del Dotto, Valentina Losacco, Ludovica Sofia Guadalupi, Cosima Damiana Calvano, David C. Chan, Valerio Carelli, Luigi Palmieri, Tommaso R.I. Cataldi

**Affiliations:** 1 Dipartimento di Chimica- Università degli Studi di Bari Aldo Moro, Bari, Italy; 2 Centro Interdipartimentale SMART- Università degli Studi di Bari Aldo Moro, Bari, Italy; 3 Dipartimento di Bioscienze, Biotecnologie e Ambiente - Università degli Studi di Bari Aldo Moro, Bari, Italy; 4 IRCCS Istituto delle Scienze Neurologiche di Bologna, Programma di Neurogenetica, Bologna, Italy; 5 Dipartimento di Scienze Biomediche e Neuromotorie, Università degli Studi di Bologna, Bologna, Italy; 6 Division of Biology and Biological Engineering, California Institute of Technology, Pasadena, CA, USA; 7 CNR-Istituto di Biomembrane, Bioenergetica e Biotecnologie Molecolari, Bari, Italy

**Keywords:** glycerophospholipids, lipidomics, mitochondria, phospholipids, phospholipids/biosynthesis, OPA1, mitofusins, mouse embryonic fibroblasts, hydrophilic interaction liquid chromatography, high resolution mass spectrometry

## Abstract

Depletion or mutations of key proteins for mitochondrial fusion, like optic atrophy 1 (OPA1) and mitofusins 1 and 2 (Mfn 1 and 2), are known to significantly impact the mitochondrial ultrastructure, suggesting alterations of their membranes’ lipid profiles. In order to make an insight into this issue, we used hydrophilic interaction liquid chromatography coupled with electrospray ionization–high resolution MS to investigate the mitochondrial phospholipid (PL) profile of mouse embryonic fibroblasts knocked out for OPA1 and Mfn1/2 genes. One hundred sixty-seven different sum compositions were recognized for the four major PL classes of mitochondria, namely phosphatidylcholines (PCs, 63), phosphatidylethanolamines (55), phosphatidylinositols (21), and cardiolipins (28). A slight decrease in the cardiolipin/PC ratio was found for Mfn1/2-knockout mitochondria. Principal component analysis and hierarchical cluster analysis were subsequently used to further process hydrophilic interaction liquid chromatography–ESI-MS data. A progressive decrease in the incidence of alk(en)yl/acyl species in PC and phosphatidylethanolamine classes and a general increase in the incidence of unsaturated acyl chains across all the investigated PL classes was inferred in OPA1 and Mfn1/2 knockouts compared to WT mouse embryonic fibroblasts. These findings suggest a reshaping of the PL profile consistent with the changes observed in the mitochondrial ultrastructure when fusion proteins are absent. Based on the existing knowledge on the metabolism of mitochondrial phospholipids, we propose that fusion proteins, especially Mfns, might influence the PL transfer between the mitochondria and the endoplasmic reticulum, likely in the context of mitochondria-associated membranes.

The field of mitochondrial medicine and biology witnessed a tremendous development over the last 30 years (1, 2). Beyond the strict domain of mitochondrial diseases related to defective oxidative phosphorylation (OXPHOS) and energy production in the form of ATP (3), mitochondria are currently recognized as the crossroad of numerous metabolic and signaling pathways displaying many interactions with other cytoplasmic structures (4). Thus, the field expanded to include diseases related to defective maintenance of mitochondrial genome (5), mitochondrial dynamics and quality control (6), and defective interactions with lysosomes and endoplasmic reticulum (ER) (7).

Mitochondria are known to retain their DNA (mtDNA), a circular genome of about 1.6 kb (8), yet the latter coordinates with the nuclear DNA (nDNA), encoding for the large majority of mitochondrial proteins (9). Mutations in either of these two genomes may lead to human disease, either rare or very rare individually, but overall representing the most frequent cause of genetic pathologies in humans (10).

Mitochondria are dynamic organelles, undergoing continuous remodeling due to fusion or fission of their membranes. This leads to a variable morphological appearance of mitochondria within cellular cytoplasm, ranging from a network of interconnected tubular mitochondria to a fragmented configuration of isolated organelles (11). Major players of mitochondrial network dynamics are optic atrophy 1 (OPA1) and mitofusins (Mfn1 and Mfn2), involved respectively in the fusion of the inner mitochondrial membrane (IMM) and of the outer mitochondrial membrane (OMM), and the dynamin-related protein 1, deputed to the fission of OMM (12, 13). Fusion favors intermixing of material, leading to the rejuvenation of membranes and enzymatic complement, as well as compensating for defective mtDNA genomes. Fission allows for the isolation of excessively deteriorated mitochondria to be targeted for autophagic removal by fusing with lysosomes (12, 13). Fission is also instrumental in segregating newly replicated mtDNA molecules, and the signaling of fission location and events implies a strict interaction with the ER (13).

Mitochondria and ER also establish areas of close membrane juxtaposition called mitochondria-associated membranes, where several different functions occur, from the exchange of calcium ions to phospholipid (PL) biogenesis and metabolism (14). The complexity of the relationship between ER and mitochondria is highlighted by the fact that ER is the major site of membrane lipid synthesis in the cell and lipids are continuously transferred/shuttled to, within, and out of mitochondria (15). This bidirectional lipid transport is fundamental for the maintenance of lipid composition of mitochondrial membranes and is also related to the fact that mitochondria themselves contribute to the synthesis of the same membrane lipids in cells (15). Furthermore, the lipid composition of the IMM is crucial for several mitochondrial functions, such as cristae formation, apoptosis, assembly of OXPHOS enzymes into supramolecular supercomplexes, and mitochondrial morphology (16). In particular, two PL classes, phosphatidic acids and cardiolipins (CLs), have been associated with the functionality of mitochondrial dynamics proteins (17, 18, 19, 20, 21). Another striking finding is the connection between Mfn 2 and maintenance of coenzyme Q levels, attributed to a decreased level of enzymes involved in the terpenoid biosynthesis pathway (22).

Overall, understanding how mitochondrial dynamics defects leading to mitochondrial diseases impinge on the mitochondrial membranes’ lipid profile remains a largely unexplored landscape. Until now, lipidomics investigations using MS have primarily focused on entire cells that lack or have reduced levels of OPA1 proteins. Chao de la Barca *et al.* used shotgun lipidomics to study the PL profiles of OPA1-deficient rat primary cortical neurons (23) and to compare OPA1-deleted mouse embryonic fibroblasts (MEFs) (24). The study revealed significant differences in the concentration of some phosphocholines (PCs), along with the corresponding lyso forms (lyso-PC), and sphingomyelins, suggesting the occurrence of PL remodeling. A similar analytical approach was adopted by Cretin *et al.* (25) to investigate the whole-cell phospholipidome of WT MEFs and of MEFs with OPA1 partially depleted by using clustered regularly interspaced short palindromic repeats (OPA1^Crispr^). The MS-based analysis revealed significant differences in the distribution of major PL classes upon OPA1 depletion and enabled the observation of changes in the distribution of specific CL species (25). In a further study, reversed-phase liquid chromatography coupled with high-resolution MS was employed to analyze lipid extracts obtained from WT and OPA1 KO MEFs, leading to the identification of 212 compounds belonging to 14 different lipid subclasses, with 69 of them exhibiting significant differences between the two types of cells (26). However, CLs could not be detected, likely because the target of investigation was the entire cellular lipidome, rather than the mitochondrial one, where CL are preferentially located.

To further explore the relationship between fusion proteins deficiency and the lipid composition of mitochondrial membranes, we adopted a different analytical approach based on hydrophilic interaction liquid chromatography (HILIC). HILIC has progressively emerged as a powerful approach for the separation of polar lipids, especially PLs, before detection based on ESI-MS (27). Notably, the retention of PL on the polar stationary phases typical of HILIC is regulated mainly by their headgroup characteristics (28, 29), thus facilitating the separation of PL classes.

In the present study, we used HILIC coupled to high-resolution Fourier-transform MS with ESI (HILIC-ESI-FTMS) to perform a comprehensive analysis of PLs in the lipid extracts obtained specifically from mitochondria of WT MEFs and MEFs knocked out either of OPA1 (OPA1^−/−^) or of Mfn1/2 (Mfn1/2^−/−^). The resulting data were subsequently processed by chemometrics to emphasize differences in the phospholipidome of the target mitochondria and have a general picture of the remodeling occurring in the absence of fusion proteins.

## Materials and Methods

### Experimental design

Three lines of MEFs, namely WT and MEFs with deleted OPA1 (OPA1^−/−^) or Mfn 1 and 2 genes (Mfn1/2^−/−^), were subjected to cell culture and three mitochondrial pellets were prepared, according to the conditions described later, to obtain three independent biological replicates for each line. Each mitochondrial pellet was then divided into three aliquots, corresponding to the same overall protein amount. Each pellet aliquot was subjected to lipid extraction and subsequent HILIC-ESI-FTMS analysis independently. Concentration values obtained for each detected PL from the analysis of the three lipid extracts referred to the same mitochondrial pellet were averaged, to account for the eventual variability related to the extraction/analysis procedure. Finally, data referred to the three different mitochondrial pellets obtained for each of the three MEF lines under study were averaged, to account for the biological variability eventually arising from the cell culture/mitochondria isolation procedure.

### Chemicals

LC-MS grade water, methanol, and acetonitrile, employed for HILIC mobile phase preparation, HPLC-grade methanol, and chloroform, used for lipid extraction, and reagent-grade ammonium acetate, used as HILIC mobile phase additive, were all purchased from Sigma-Aldrich (Milan, Italy). The EQUISPLASH® LIPIDOMIX® quantitative MS internal standard, including 13 deuterated lipids at a concentration of 100 μg/ml each, was purchased from Avanti Polar Lipids (Alabaster, AL) and the following species among them were adopted as internal standards for quantitative determinations referred to the respective PL classes: 1-pentadecanoyl-2-oleoyl(d7)-sn-glycero-3-PC for PC; 1-pentadecanoyl-2-oleoyl(d7)-sn-glycero-3-phosphoethanolamine for phosphatidylethanolamine (PE); 1-pentadecanoyl-2-oleoyl(d7)-sn-glycero-3-phosphoinositol for phosphoinositol (PI). The standard of 1′,3′-bis[1,2-dimyristoyl-sn-glycero-3-phospho]-glycerol (CL 14:0/14:0/14:0/14:0) was purchased from Sigma-Aldrich (Milan, Italy) and used as an internal standard for the quantification of CLs, since its absence in mitochondrial lipid extracts was preliminarily ascertained. The following standard PLs: 1-myristoyl-2-palmitoyl-sn-glycero-3-PC, 1,2-dipalmitoleoyl-sn-glycero-3-PC, 1-stearoyl-2-linoleyl-sn-glycero-3-PC, 1-(1Z-octadecenyl)-2-linoleoyl-sn-glycero-3-PC, 1,2-dimiristoyl-sn-glycero-3-phosphoethanolamine, 1-(1Z-octadecenyl)-2-oleoyl-sn-glycero-3-phosphoethanolamine, 1,2-distearoyl-sn-glycero-3-phosphoinositol, 1-pentadecanoyl-2-oleoyl-sn-glycero-3-PI, 1′,3′-bis[1-palmitoyl-2-oleoyl-sn-glycero-3-phospho]-glycerol (CL 16:0/18:1/16:0/18:1), and 1',3′-bis[1,2-dioleoyl-sn-glycero-3-phospho]-glycerol (CL 18:1/18:1/18:1/18:1), used to check the electrospray ionization yields of different compounds belonging to the same PL class following HILIC separation, were purchased from Avanti Polar Lipids (Alabaster, AL).

### Cells, culture conditions, and mitochondria isolation

WT, OPA1^−/−^, and Mfn1/2^−/−^ MEFs were cultured at 37°C in a humidified atmosphere including 5% CO_2_ and in a high glucose DMEM (Euroclone, Milan, Italy) supplemented with 10% fetal bovine serum (Euroclone, Milan, Italy), 50 U of penicillin G/ml, 50 μg/ml of streptomycin sulfate, and 2 mM of L-glutamine (Sigma-Aldrich, Milan). Cell counting was performed using the Scepter™ Automated Cell Counter (Sigma-Aldrich, Milan), according to the manufacturer's instructions, in parallel to trypan blue visualization, adopted to exclude significant differences in live or dead trypsinized cells ratio among the tested cells.

Mitochondria from MEF cell lines were isolated using Dounce homogenization, following the protocol of the mitochondria isolation kit for cultured cells (product code 89874, purchased from Thermo Fisher Scientific, Rodano, Italy), slightly modified to obtain a more purified fraction of mitochondria, with more than 50% reduction of lysosomal and peroxisomal contaminants. As previously reported (30), the use of Dounce homogenization is expected to reduce nuclear contaminations during mitochondria isolation, compared to differential centrifugation.

In particular, Dounce homogenization was performed after incubating cells (20 × 10^6^) with 800 μl of the kit reagent A for 2 min on ice, then 800 μl of reagent C were added, and the suspension was centrifugated for 10 min at 700 *g* and 4°C. The resulting supernatant was centrifugated for 15 min at 3,000 *g* and 4°C, and then the mitochondrial pellet was retrieved and washed with 500 μl of reagent C. Finally, centrifugation at 12,000 *g* at 4°C was performed for 5 min, and the resulting pellet was collected. Protein concentration related to mitochondria was determined using the BCA protein assay reagent (product code 23227, Thermo Fisher Scientific, Rodano, Italy).

### Western blotting and enzymatic assays

To evaluate the purity of mitochondrial samples, the immunodetection of citrate synthase (CS) and β-actin was carried out in whole MEFs and the mitochondrial fractions derived from them. The cells were lysed in RIPA buffer (product code 89900, Thermo Fisher Scientific, Rodano, Italy) and 20 μg of total proteins relative to whole cells or mitochondria obtained from them were treated with 10 mM Tris/HCl pH 6.8, 2% SDS, 5% β-mercaptoethanol. Afterward, they were subjected to 15% SDS-polyacrylamide gel electrophoresis and subsequently transferred on nitrocellulose membrane. Western blot analysis was performed using antibodies against CS (MA5-17264, Invitrogen, Waltham, MA) and β actin (MA5-15739, Invitrogen, Waltham, MA). Antigen–antibody complexes were detected using anti-mouse IgG-coupled horseradish peroxidase (product code 31430, Thermo Fisher Scientific, Waltham, MA). Densitometric analysis of the relative bands was accomplished by using the Image Lab™ Touch software (Bio-Rad Laboratories, Hercules, CA).

CS activity was evaluated in the cellular lysate and in mitochondrial samples considering 30 μg of proteins for both. The evaluation was performed by measuring the absorption at 412 nm related to thionitrobenzoic acid, which, in the presence of saturating concentrations of substrates acetyl-CoA, oxaloacetate, and dithionitrobenzoic acid, is a function of CS activity (31).

### Lipid extraction from MEF mitochondria

Lipid extraction from mitochondria was performed using a slightly modified version of the Bligh and Dyer) protocol (32). Specifically, mitochondrial pellets obtained from each MEF line were resuspended into 2.4 ml of LC-MS water at room temperature, then the suspension was divided into three 800 μl aliquots, each subjected to lipid extraction as follows: 3 ml of a methanol/chloroform 2:1 (v/v) mixture were added; the resulting suspension was vortexed for 1 min and then left quiescent in the dark and in an ice bath for 1 h. At the end of this step, the suspension was centrifuged at 4,500 *g* for 10 min, and the supernatant was quantitatively withdrawn. Equal volumes (1.25 ml) of water and chloroform were added and, after vortexing for 1 min, the resulting mixture was centrifuged at 4,500 *g* for 10 min, to facilitate phase separation. A 1.5 ml volume of the underlying, chloroform-rich, phase was subsequently collected and evaporated to dryness under a gentle nitrogen flux. Afterward, the solid residue was redissolved in 100 μl of a methanol/chloroform 2:1 (v/v) mixture and the resulting solution was transferred into a screw-cap vial, whose headspace was saturated with nitrogen (to minimize lipid oxidation) before storage at −20°C until HILIC-ESI-FTMS analysis was performed. To estimate the concentrations of PL species recognized in a specific class, starting from the MS response of the respective internal standard, an aliquot of the final extract was spiked with appropriate volumes of the EQUISPLASH® LIPIDOMIX® internal standard and a 100 μg/ml solution of the CL 56:0 standard, before being subjected to HILIC-ESI-FTMS analysis. The spiking was performed to reach a 3 μg/ml final concentration for each standard lipid in the final extract.

### HILIC-ESI-MS instrumentation and operating conditions

HILIC-ESI-FTMS analyses of lipid extracts obtained from MEF mitochondria were performed using an Ultimate 3000 HPLC quaternary chromatographic system coupled to a Q-Exactive high-resolution Fourier-transform quadrupole-Orbitrap mass spectrometer (Thermo Fisher Scientific, West Palm Beach, CA) equipped with a heated electrospray ionization (HESI) source. Chromatographic separations were performed using an Ascentis Express HILIC column (15 cm length, 2.1 mm internal diameter) packed with core-shell 2.7 μm silica particles (Supelco, Bellefonte, PA) and preceded by a guard column (2 cm length, 2.1 mm internal diameter), packed with the same type of particles. A 0.3 ml/min flow rate, a 5 μl sample volume and a binary elution gradient based on an acetonitrile/water (97:3 v/v) mixture as phase A and a methanol/water (90:10 v/v) mixture as phase B, both containing ammonium acetate 2.5 mM, were adopted for lipid separation. The elution program was the following: 0–10 min) linear increase of B from 2 to 20%; 10–15 min) linear increase of B from 20 to 50%; 15–20 min) isocratic at 50% B; 20–25 min) return to 2% B; 25–30 min) column reconditioning at 2% B.

The parameters of the HESI interface and of the ion optics of the Q-Exactive spectrometer adopted for FTMS analyses were set as follows: sheath gas flow rate, 35 a.u.; auxiliary gas flow rate, 15 a.u.; spray voltage, ± 2.5 kV for positive/negative ion acquisitions; capillary temperature, 320°C; and S-lens RF level, 100. During each run FTMS full scan measurements were performed by operating the mass spectrometer at its maximum resolving power (140,000 at *m/z* 200) using a 100–2,000 *m/z* interval; the Orbitrap fill time was set to 200 ms and the automatic gain control level was set to 1 × 10^6^. Additionally, all ion fragmentation (AIF) measurements (alternated to full scan ones) were performed during the same runs, that is, a systematic fragmentation was affected in the higher-energy collisional dissociation cell of the Q-Exactive spectrometer on ions generated in the HESI source, using a 35% normalized collisional energy. The resulting product ion spectra were acquired in a 150–800 *m/z* interval, using the maximum available resolving power. AIF acquisitions were employed to recognize chromatographic bands related to specific PL classes through the extraction of currents related to class-specific product ions (vide infra). The spectrometer was calibrated daily by infusing, at a 5 μl/min flow rate, calibration solutions provided by the instrument manufacturer for positive or negative polarity acquisitions. As a result, a mass accuracy always better than 5 ppm was achieved.

### HILIC-ESI-FTMS data processing

The processing of HILIC-ESI-FTMS data was focused on the four major PL classes detected in mitochondrial extracts, that is, PC, PE, PI, and CL. Firstly, FTMS spectra were averaged in retention time intervals where bands related to those classes were eluted, using positive ion acquisitions for PCs and negative ion ones for PEs, PIs, and CLs (vide infra). Extracted ion current chromatograms obtained from HILIC-ESI-AIF-FTMS datasets for PL class-specific product ions, related to the PL headgroups, were employed to recognize those intervals. Specifically, the positively charged product ion with exact *m/z* 184.0733 ([C_5_H_15_NO_4_P^+^]), corresponding to the PC ion, was adopted for PCs; the negative ion with exact *m/z* 196.0380 ([C_5_H_11_NO_5_P^-^]), corresponding to the deprotonated form of dehydro-glycerophosphoethanolamine, was used for PEs; the *m/z* 241.0119 ion ([C_6_H_10_O_8_P^-^]), corresponding to deprotonated phospho-dehydroinositol, was selected for PIs, and the *m/z* 152.9958 ion ([C_3_H_6_O_5_P^-^]), corresponding to dehydroglycerophosphate, was adopted for CLs.

PL class related averaged FTMS spectra, which can be considered equivalent to shotgun MS data for specific PL classes, were processed using the *Alex*^*123*^ *eXtractor* routine, included in the *Alex*^*123*^ software, freely available on the World Wide Web (www.mslipidomics.info) (33). This step enabled the recognition of the sum compositions of the detected PL. Following the shorthand notation proposed by Liebisch *et al.*, sum compositions for diacylic PL were indicated as C:D, with C and D representing, respectively, the total numbers of carbon atoms and double C=C bonds on the two side chains (34). Since also alk(en)yl-acyl PC and PE were identified in MEF mitochondria lipid extracts, the PC O and PE O general notations, followed by the sum composition of the two side chains, were adopted for them, respectively. Sum compositions were inferred through the comparison between accurate experimental *m/z* values referred to monoisotopic (M+0) singly charged ions for PCs, PEs, and PIs, and to monoisotopic doubly charged ions for CLs, and those enclosed in a target list preliminarily generated using the *Alex*^*123*^ *Target List Generator* routine. Based on the available mass accuracy, a match was considered acceptable when the difference between the accurate and theoretical *m/z* values was lower than 0.05 units. The recognized PL species for each class were subsequently subjected to an intensity-based filtering, that is, to the selection of species whose monoisotopic ions exhibited an intensity higher than 1% of that of the base peak in the PL class-related spectrum, considering only endogenous species. Sum compositions and signal intensities for monoisotopic peaks of selected species were subsequently subjected to *type-I* and *type-II* isotopic corrections using the LIPIC software, recently developed in our laboratory (35). *Type-I* correction was performed to obtain a signal intensity accounting for the entire isotopic pattern for each PL species, starting from the intensity of the respective monoisotopic peak and the knowledge of its molecular formula. *Type-II* correction was performed to account for the spectral interference eventually occurring between peak signals referred to the M+0 isotopolog of a specific PL and the M+2 isotopolog of another PL of the same class including a further C=C bond along its side chain. Due to the remarkable extent of coelution of compounds belonging to the same PL class, this kind of interference can be quite important in FTMS spectra of PL classes separated by HILIC.

Corrected peak intensities for PL species in a class were subsequently employed to calculate relative abundances by dividing each of them by their total. Relative abundance values obtained for each PL after the analysis of the three extraction replicates resulting from each mitochondrial preparation were averaged and the corresponding relative standard deviations were calculated. Peak intensities for which a relative standard deviation value higher than 20% was achieved were not considered for subsequent chemometrics elaboration referred to all cell lines, so that only species with relatively reproducible responses could be included in the latter. Corrected peak intensities obtained for PL species after the analysis of mitochondrial lipid extracts preliminarily spiked with deuterated lipids and CL 56:0 were employed to retrieve an estimate of concentrations of those species in each sample. The estimation was based on the ratio between the peak intensity subjected to *type-I*/*type-II* correction referred to each endogenous PL and the one obtained for the PL adopted as the internal standard for the same class, added at a known concentration to the lipid extract before analysis. Notably, the estimate of PL concentrations based on a single internal standard per class relies on the assumption that molecules belonging to the same class ionize similarly in the adopted conditions, since ionization is related to the same headgroup and occurs in the presence of very similar compositions of the mobile phase, due to the HILIC separation. The validity of this approach, well-established in the context of MS-based lipidomics (36), was recently confirmed for PC in our laboratory (37).

A further control of the similarity of ionization yields for structurally different PCs, PEs, PIs, and CLs under the conditions adopted for HILIC-ESI-FTMS analysis of mitochondrial lipid extracts was performed during the present study. In particular, the intensity of the peak referred to the respective M+0 isotopolog, retrieved from the mass spectrum averaged under the HILIC band of the respective class for each species in the following groups: PC 14:0/16:0, 16:1/16:1, 18:0/18:2 and P-18:0/18:1; PE 14:0/14:0 and P-18:0/18:1; PI 15:0/18:1 and 18:0/18:0; CL 14:0/14:0/14:0/14:0, 16:0/18:1/16:0/18:1 and 18:1/18:1/18:1/18:1 was subjected to type-I correction and calibrated versus concentration in a 0.01–15 μM range (six levels were adopted for each calibration). As evidenced in Supplemental Fig. S1 of the Supplementary materials, no statistically significant difference was observed for the calibration line slopes obtained for PLs belonging of the same class, despite differences occurring in the length and/or the unsaturation degree of their acyl chains and even when diacyl and alkenyl/acyl species were compared, as in the case of PCs and PEs.

Concentrations of single species in a class obtained as described before were subsequently employed also to calculate the total concentrations of PCs, PEs, PIs, and CLs in each analyzed sample. The latter allowed the estimation of concentration ratios between selected PL classes, useful for the comparison between different MEF lines.

### Statistical analysis and chemometrics

The comparison of average concentration ratios between PL classes obtained for the three types of mitochondria under study was performed using a post hoc Tukey test. Relative abundance data referred to mitochondrial PCs, PEs, PIs, and CLs, obtained for three biological replicates of WT, OPA1^−/−^, and Mfn1/2^−/−^ MEFs, according to the procedure described previously, were processed using principal component analysis (PCA) and hierarchical cluster analysis (HCA) with heatmap. The *statistical analysis (one factor)* module of the *MetaboAnalyst* website (38) (freely accessible at the World Wide Web address https://www.metaboanalyst.ca) was used for this aim. Both PCA and HCA were based on autoscaled data; HCA was performed using Euclidean distances and the average linkage agglomeration algorithm. Biplots and dendrograms with heatmaps were generated after data processing based on PCA and HCA, respectively. In the case of PCA, 95% confidence ellipses could be also drawn in the biplots, since samples were classified according to the type of cell lines in Excel files used to upload input data.

## Results

### Assessment of mitochondrial purity

**Fig. 1:**
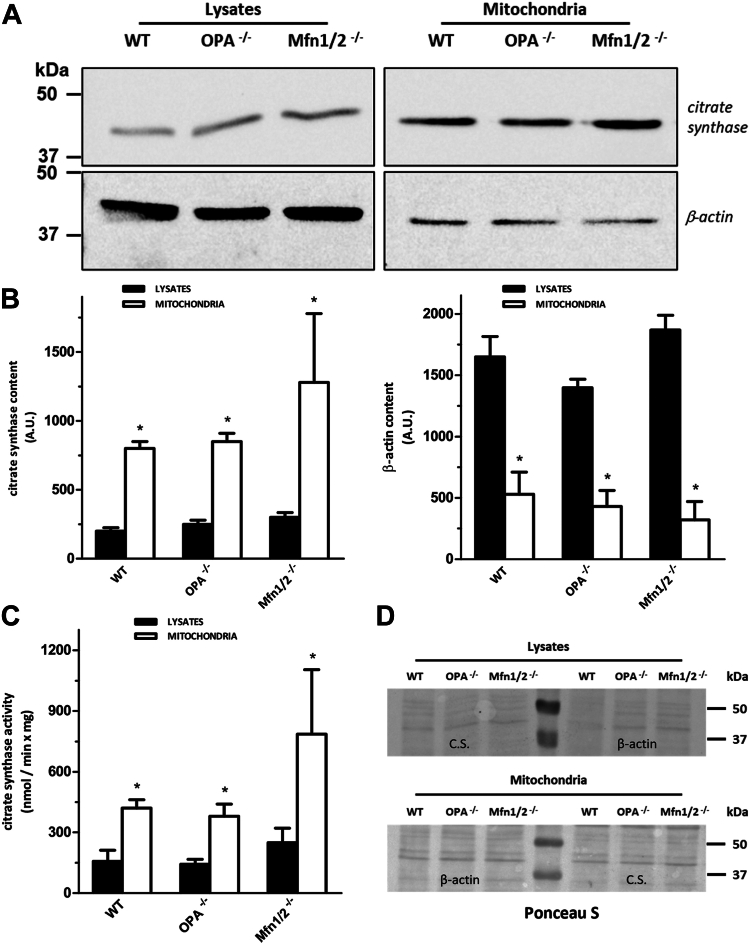
Assessment of the purity and enrichment of mitochondria obtained from MEFs. A: A representative Western blot of three independent experiments performed on total cell extract and purified mitochondria relative to WT, OPA1^−/−^, and Mfn1/2^−/−^ MEF cell lines. Ten micrograms of lysates and purified mitochondria were separated on 12% polyacrylamide gels and transferred onto nitrocellulose membranes. Western blot analysis was performed using antibodies against a mitochondrial marker, citrate synthase, and a cytosolic protein, β-actin. Subsequently, the same membranes were incubated with secondary antibodies host in mouse and rabbit, respectively, and conjugated with HRP. B: Citrate synthase and β-actin content were quantified by densitometric analysis of three independent experiments of Western blot. Significant differences in lysates and mitochondria for each cell line are marked with ∗ (*P* < 0.01, *t*-student). C: Citrate synthase activity, expressed as initial rate, measured in cell lysate and relative mitochondria. Average and SD of three independent experiments (∗*P* < 0.01). D: As loading control before Western blot assay, the membranes relative to lysates and purified mitochondria were stained with red Ponceau S, cut, and blotted for the protein indicated (citrate synthase; β-actin). HRP, horseradish peroxidase; MEF, mouse embryonic fibroblast; Mfn 1/Mfn 2, mitofusin 1/mitofusin 2; OPA1, optic atrophy 1.

The purity and the enrichment of mitochondria from whole MEF cells were preliminarily assessed by Western blot analysis measuring cytosolic (β-actin) and mitochondrial (CS) marker proteins. A representative Western blot of the total extract and enriched mitochondrial fraction of MEFs is reported in Fig. 1A (details on the procedure adopted to obtain cropped gel images in this figure are reported in Supplemental Fig. S2 of the Supplementary materials). Densitometric analysis of the bands detected in three different experiments showed that the mitochondrial fraction (M) derived from WT and OPA1^−/−^ cells was enriched about 3-fold in terms of CS compared to the relative cellular lysate (L) and this increase was more evident when lysate and mitochondria relative to Mfn1/2^−/−^ MEFs were compared. However, no significant differences in CS content was measured in control mitochondria compared to OPA1^−/−^ and Mfn1/2^−/−^ ones, respectively. Furthermore, cytosolic contamination in terms of β-actin was very low in mitochondrial fractions of the three cell lines examined here. By contrast, β-actin was more abundant in the lysate of each line compared to the relative mitochondrial fraction (Fig. 1B). Firstly, this result confirms the purity of mitochondria used for lipidomic analysis, but also supports the mitochondrial localization of a pool of β-actin, as demonstrated by Xie and colleagues (39). Moreover, the activity of CS, an enzyme of the tricarboxylic acid cycle, was measured. As shown in Fig. 1C, specific activity for this enzyme was significantly increased upon purification of mitochondria from cells for all three cell lines examined, confirming the results obtained by Western blot analysis.

### Characterization of PCs, PEs, PIs, and CLs in mitochondrial lipid extracts of MEFs

**Fig. 2:**
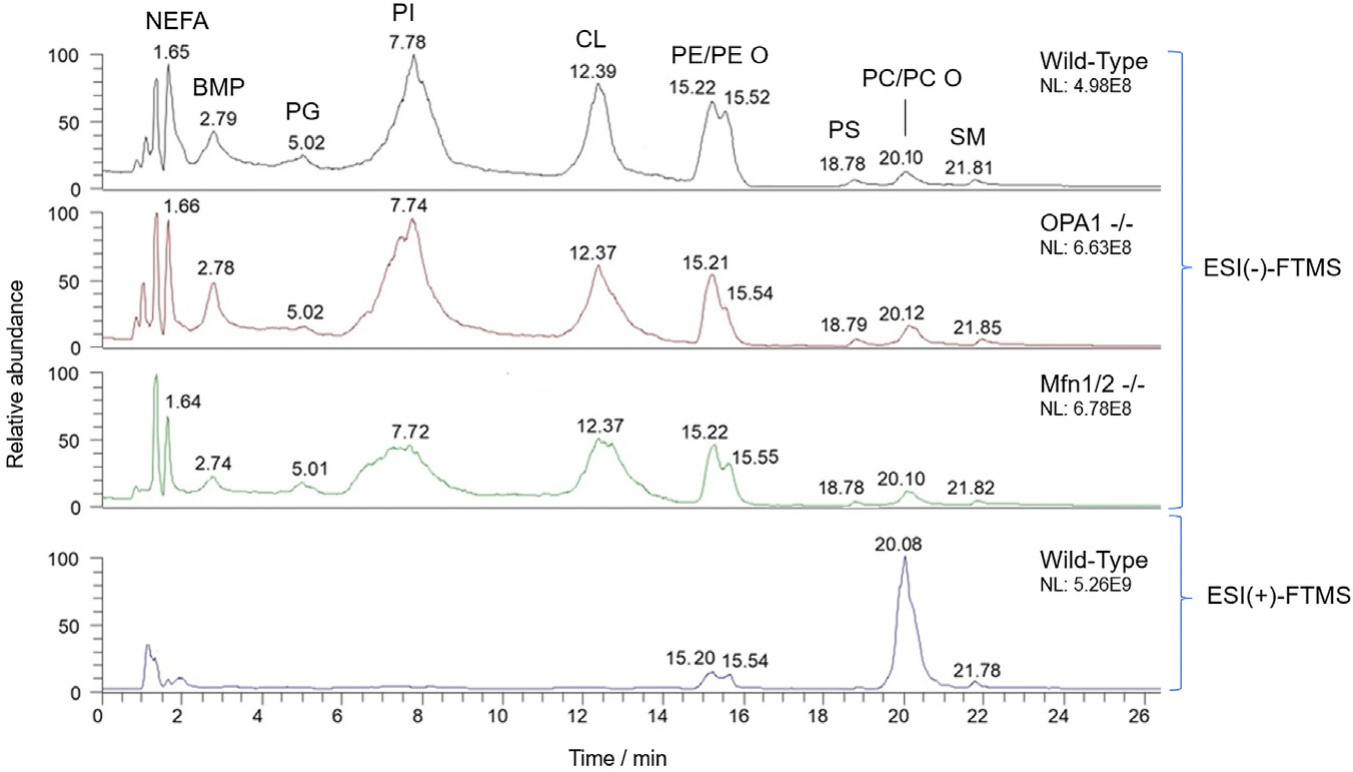
HILIC-ESI-FTMS provides information on the occurrence of major lipid classes in MEF mitochondria. Comparison between the total ion current (TIC) chromatograms resulting from the HILIC-ESI (−)/(+)-FTMS analysis of lipid extracts of mitochondria isolated from WT, OPA1^−/−^ and Mfn1/2^−/−^ MEFs. Legend for identified lipid classes, in order of retention time: nonesterified fatty acids (NEFAs), bis-monoacylglycerophosphates (BMPs), phosphatidylglycerols (PGs), phosphatidylinositols (PIs), cardiolipins (CLs), phosphatidylethanolamines (PEs), phosphatidylserines (PSs), phosphatidylcholines (PCs), and sphingomyelins (SMs). PE O and PC O indicate alk(en)yl/acyl PE and PC, respectively. NL, normalization levels used to calculate relative abundances. FTMS, Fourier-transform mass spectrometry; HILIC, hydrophilic interaction liquid chromatography; MEF, mouse embryonic fibroblast; Mfn 1/Mfn 2, mitofusin 1/mitofusin 2; OPA1, optic atrophy 1.

As exemplified in Fig. 2, where HILIC-ESI(−)-FTMS total ion chromatograms (TICs) referred to lipid extracts of mitochondria obtained from WT, OPA1^−/−^, and Mfn1/2^−/−^ MEFs are reported, HILIC provided a good class-selective separation also in the case of mitochondrial PLs. For the sake of comparison, the HILIC-ESI(+)-FTMS TIC trace referred to the lipid extract of WT MEF mitochondria is also shown in the figure. As apparent, well-defined bands were observed in the TIC traces and subsequently assigned to seven PL classes, that is, phosphatidylglycerols, phosphatidylinositols (PIs), CL, diacylic (PE), and alk(en)ylic/acylic PEs, phosphatidylserines (PS), diacylic (PC) and alk(en)ylic/acylic (PC O) phosphatidylcholines, and SM, in order of retention time. Early eluting bands referred to nonesterified fatty acids (NEFAs) and bis-monoacylglycerophosphates were recognized in the same type of chromatogram. Not surprisingly, PCs were detected much better in the HILIC-ESI(+)-FTMS ion TIC trace since they are preferentially ionized as positive ions. A preliminary comparison of TIC traces (Fig. 2) indicates that mitochondria obtained from the three MEF lines under investigation had similar PL profiles. In accordance with information previously reported on rat liver mitochondria (39), PS, SM, and phosphatidylglycerol appeared as minor classes. Based on the PL profiles inferred from HILIC-ESI-FTMS TIC traces, the present investigation focused on PC, PE, PI, and CL classes, starting from the evaluation of the FTMS spectra averaged in retention time intervals corresponding to the respective chromatographic bands. Examples of those spectra, referred to WT MEF mitochondrial lipid extracts, are reported in Supplemental Fig. S3 (for PCs and PEs) and Supplemental Fig. S4 (for PIs and CLs) of the Supplementary materials.

Both PC and PE classes were characterized by the occurrence of diacylic and alk(en)ylic/acylic species (*i.e.*, PC O and PE O), with a remarkable variability in their sum compositions. Indeed, the latter included all the possible even numbers of carbon atoms between 30 and 40 for PCs and between 32 and 42 for PEs, with major PCs exhibiting a total of 32–36 total carbon atoms and 1–3 C=C bonds on their side chains. PE/PE Os with 36 or 38 carbon atoms and up to 7 C=C bonds, indicating a remarkable degree of side chain unsaturation, were found to be the most relevant species in their class (see Supplemental Fig. S3). As reported in Supplemental Fig. S4, a limited structural variability was inferred for PI and CL classes. Indeed, no alk(en)ylic-acylic species were detected for them, as expected; moreover, major diacylic species including 36 or 38 carbon atoms and up to 5 C=C bonds were observed for PIs. In the case of CLs, clusters of sum compositions including 64 to 72 atoms and up to 7 C=C bonds were found. Interestingly, PIs and CLs were the only major PLs in MEF mitochondria for which species with odd numbers of total carbon atoms on the side chains (35–37 and 67, 69, and 71, respectively) were also detected (see Supplemental Fig. S4).

It is important to highlight that sum compositions shown in Supplemental Figs. S3 and S4 could be accurately determined only after accounting for *type-II* isotopic interferences. The latter are typical of PL class-specific spectra obtained through spectral averaging under the corresponding HILIC bands. The partial coelution of PL species of the same class differing by just a C=C bond (*i.e.*, by 2 Da in the nominal molecular mass) is rather frequent in this case and may determine noticeable anomalies in the *m/z* values observed for peak signals subjected to the interference. Just to cite an example, the peak signal referred to the monoisotopic peak (M+0 isotopolog) of PC 32:0 (experimental *m/z* 734.5675) was subjected to spectral interference by the peak related to the M+2 isotopolog of PC 32:1 (experimental *m/z* for the corresponding M+0 isotopolog: 732.5531, Supplemental Fig. S3). As a result, its intensity was artificially increased and its *m/z* value appeared less accurate than expected; indeed, the shifts calculated between the theoretical and the experimental *m/z* values were −0.0007 and −0.0019 for PC 32:1 (not suffering from any *type-II* interference) and 32:0, respectively. In the case of PEs and CLs the effect was widespread, with severe *type-II* interferences occurring in all the clusters of peak signals detected in the spectrum. The use of the LIPIC software (35), recently developed in our laboratory, enabled a careful correction of these interferences and a reliable assignment of sum compositions and MS responses to PL species.

As evidenced in Supplemental Fig. S5 of the Supplementary materials, showing a typical FTMS spectrum averaged under the NEFA chromatographic band obtained for the WT MEF mitochondria lipid extracts, sum compositions observed for major PLs could be accounted for by considering the most relevant free fatty acids detected in the mitochondrial lipid extracts. While pinpointing the exact structure of individual alk(en)yl or acyl chains of PLs was beyond the scope of this study, data on NEFAs suggested combinations of side chains with 16 or 18 carbon atoms and 0–2 C=C bonds to occur in major PCs, PEs, PIs, and CLs. Interestingly, longer chain (>20) and highly unsaturated fatty acids with an even number of carbon atoms were also detected (see the vertically magnified portion of the spectrum in Supplemental Fig. S5), although with a lower abundance. Specifically, 20:x and 22:x NEFA, with x up to 6, prevailed among them and could be considered as involved in the occurrence of species like PCs, PEs, and PIs with 38–40 total carbon atoms and a high degree of unsaturation.

Once sum compositions were assessed and *type-II* corrections were performed, the peak signal intensities of the M+0 isotopolog of PLs detected in the four target classes were employed to calculate the intensity referred to the respective entire isotopic pattern, that is, to perform the *type-I* correction, using the LIPIC software (35). At the end of this procedure, the intensity related to each species in a PL class could be exploited to estimate its concentration in the mitochondrial lipid extract of a specific MEF sample. The estimate was based on the comparison with the intensity of a deuterated PL of the same class for PCs, PEs, and PIs, and of CL 56:0, not occurring in mitochondrial extracts, for CLs. These compounds were added to each lipid extract as internal standards just before the analysis. The reliability of this procedure relies on the already discussed assumption that PL species belonging to the same class and eluting in a short retention time interval, which is the typical scenario of the HILIC separation of PLs, exhibit comparable electrospray ionization yields.

**Fig. 3:**
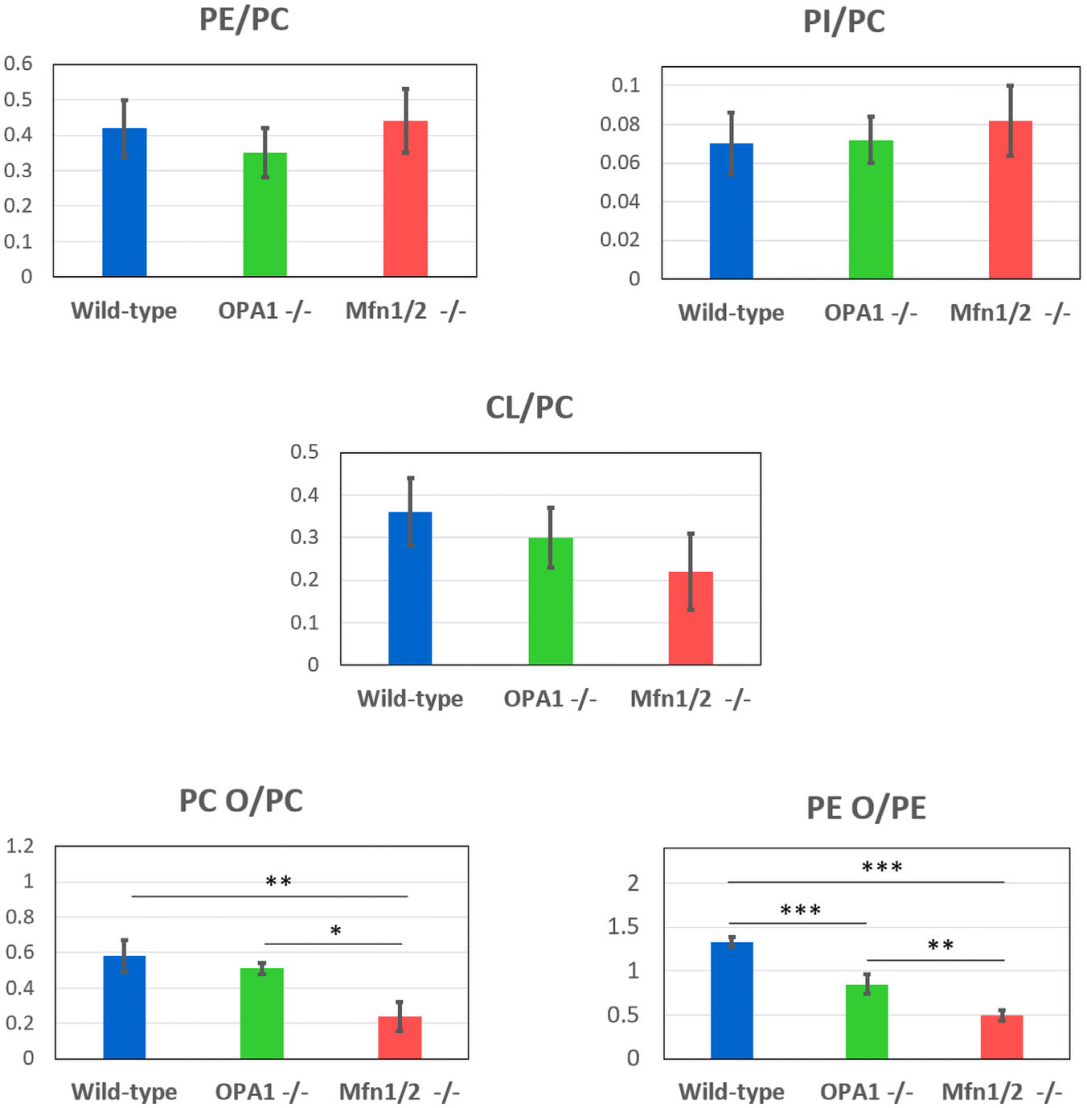
Concentration ratios between PL classes or subclasses reveal a decrease in alk(en)ylic/acylic PC and PE in mitochondria of OPA1^−/−^ and Mfn1/2^−/−^ MEFs. Concentration ratios between PE, PI, and CL, and the PC class, considered as reference, and between PC O and PE O and the respective diacylic counterparts, resulting from the HILIC-ESI-FTMS analysis of mitochondrial lipid extracts of WT, OPA1^−/−^ and Mfn1/2^−/−^ MEFs. Average values and standard deviations observed for three biological replicates are reported for each cell line. Statistically significant differences, as inferred using a Tukey Honestly Significant Difference test, are evidenced in the case of PC O/PC and PE O/PE ratios: ∗*P* < 0.01; ∗∗*P* < 0.005; ∗∗∗*P* < 0.001. CL, cardiolipin; FTMS, Fourier-transform mass spectrometry; HILIC, hydrophilic interaction liquid chromatography; MEF, mouse embryonic fibroblast; Mfn 1/Mfn 2, mitofusin 1/mitofusin 2; OPA1, optic atrophy 1; PC, phosphatidylcholine; PE, phosphatidylethanolamine; PI, phosphatidylinositol; PL, phospholipid.

Concentrations of PCs, PEs, PIs, and CLs corresponding to specific sum compositions were used to estimate the total concentration of a PL class in each lipid extract and the relative abundance of species belonging to each class. To compare the concentrations of different PL classes, PCs were used as the reference since they were the most abundant PLs. The average values and standard deviations (estimated for three biological replicates for each cell line) of the concentration ratios between PL classes (or subclasses) are reported in Fig. 3 as bar plots. As evidenced in the figure, no statistically significant differences could be inferred between WT, OPA1^−/−^, and Mfn 1/2^−/−^ mitochondria in terms of PE/PC, PI/PC, and CL/PC ratios. A progressive decrease in the CL/PC average ratios was just observed when comparing WT with OPA1^−/−^ and Mfn 1/2^−/−^ mitochondria. This outcome suggests that the absence of OPA1 and Mfn 1 and 2 did not significantly affect the overall profile of major PL classes in MEF mitochondria. Notably, the CL/PC ratios estimated for WT and for OPA1^−/−^ and Mfn 1/2^−/−^ mitochondria of MEFs (0.21–0.36, see Fig. 3) were comparable with the one (0.32) estimated for mitochondria of rat liver cells (see Ref. (40) and markedly higher than the one estimated for the ER of the latter (0.017). They were also much higher than the CL/PC ratio (0.023) estimated from PL data reported in the literature for the nuclear membrane of rat liver cells (41). These findings further confirm the limited, if any, occurrence of ER or nuclear membrane contamination in the mitochondrial samples analyzed during this study.

The observed stability of the PL profile of MEF mitochondria even in the absence of key proteins for mitochondrial fusion is a relevant finding, especially if compared with the one obtained for concentration ratios between alk(en)ylic/acylic and diacylic species both for PCs (PC O/PC) and for PEs (PE O/PC). As evidenced in Fig. 3, a significant decrease was observed for the PC O/PC ratio in Mfn1/2^−/−^ mitochondria compared to WT (*P* < 0.005) and to OPA1^−/−^ (*P* < 0.01) ones. A progressive remarkable decrease was observed for the PE O/PE ratio when comparing WT mitochondria to OPA1^−/−^ and MFn1/2^−/−^ ones (*P* < 0.001 in both cases). The PE O/PE ratio was found to be also statistically lower in MFn1/2^−/−^ mitochondria than OPA1^−/−^ ones (*P* < 0.005).

A deeper insight into the evolution of the major classes of the mitochondrial phospholipidome related to the absence of these proteins was undertaken in the subsequent step of this study using chemometrics. The relative abundances of specific sum compositions detected for PCs, PEs, PIs, and CLs were used as input variables for this analysis.

### Evaluation of differences in the PC, PE, PI, and CL intraclass profiles of MEF mitochondria by chemometrics

#### Phosphatidylcholines

**Fig. 4:**
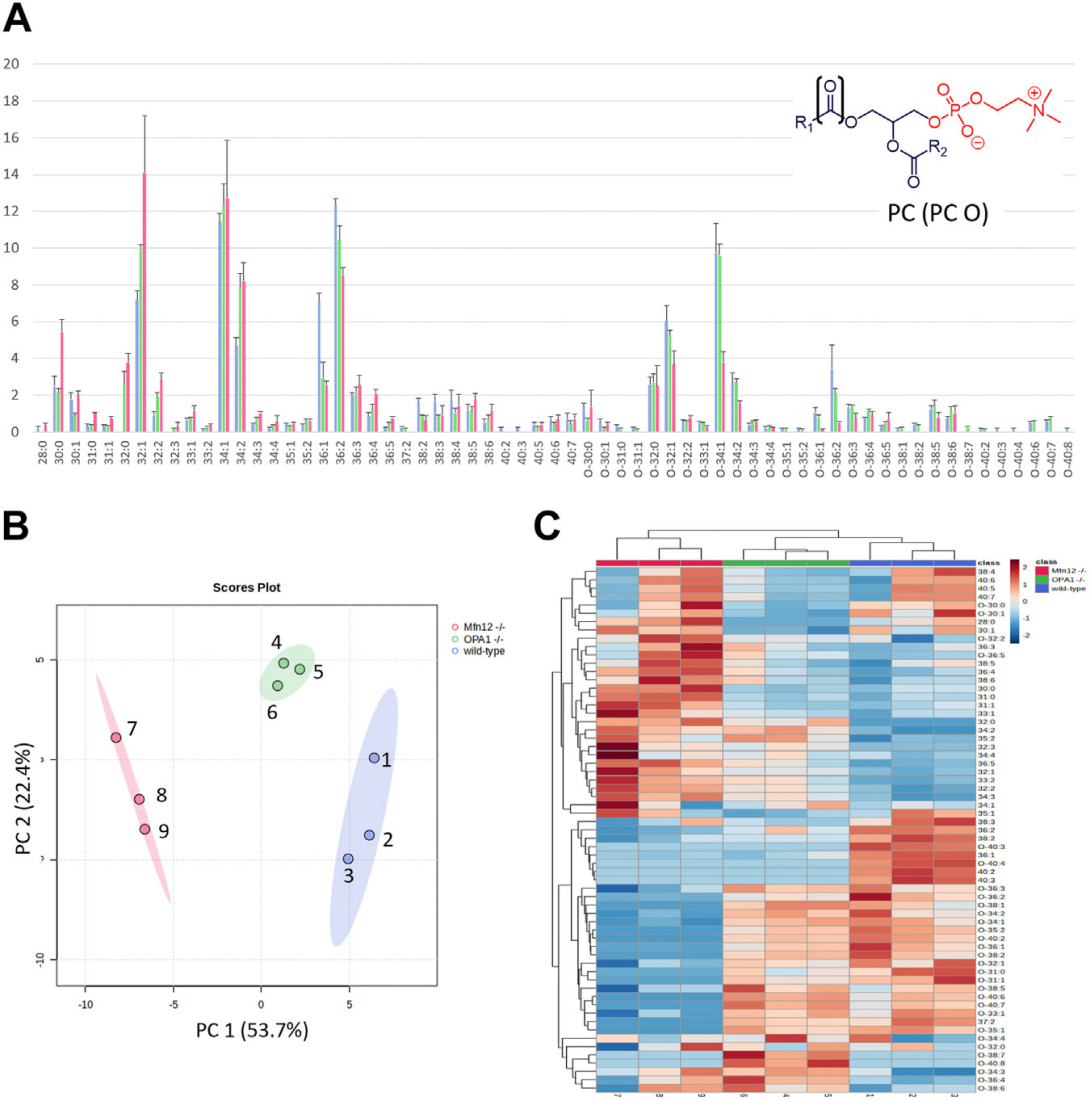
Chemometrics based on the intra-class profile of PCs and PC Os enables a distinction between mitochondria of WT, OPA1^−/−^, and Mfn1/2^−/−^ MEFs. A: Intraclass abundance profile for PC and PC O obtained after the HILIC-ESI(+)-FTMS analysis of mitochondrial lipid extracts referred to WT (light blue columns), OPA1^−/−^ (green columns), and Mfn1/2^−/−^ (red columns) MEFs. Average values for three biological replicates are reported; error bars represent standard deviations. B: Score plot referred to the first two principal components obtained after PCA based on intraclass abundances of PC and PC O, including 95% confidence regions for each type of sample. C: Sample and variable dendrograms with heatmap resulting from the HCA based on the same input data. Autoscaled values were adopted in both cases; Euclidean distances and the average linkage agglomeration algorithm were adopted for HCA. FTMS, Fourier-transform mass spectrometry; HCA, hierarchical cluster analysis; HILIC, hydrophilic interaction liquid chromatography; MEF, mouse embryonic fibroblast; Mfn 1/Mfn 2, mitofusin 1/mitofusin 2; OPA1, optic atrophy 1; PC, phosphatidylcholine; PCA, principal components analysis.

The profile of PC species in the three types of MEF mitochondria under study is reported in Fig. 4A, where vertical bars represent average percentual abundances (n = 3) of PC and PC O species. As apparent, diacylic PCs (33 sum compositions detected) were generally more abundant than PC Os (30 sum compositions) in Mfn1/2^−/−^ mitochondria (see red-colored bars), whereas more subtle differences were observed between mitochondria of WT and OPA1^−/−^ MEFs. Based on the observed variabilities, only a few species exhibited statistically different abundances across the three types of samples. For example, PC 32:1 abundance was significantly increased in the order WT < OPA1^−/−^ < Mfn1/2^−/−^, whereas PC 36:2 showed an opposite trend. On the other hand, given a specific total number of carbon atoms on the two side chains, diacylic or alk(en)ylic-acylic PC with a higher number of C=C bonds became progressively more abundant, in relative terms, in OPA1^−/−^ and Mfn1/2^−/−^ mitochondria WT ones.

Chemometrics were employed to emphasize these trends and verify if general differences in the PC profiles occurred between the three types of mitochondria under study. Indeed, a clear separation between them was observed in the score plot for the first two principal components arising from PCA based on PC relative abundances, accounting for 76.1% of total variance (see Fig. 4B). A clear clustering of the three types of samples was observed also in the sample dendrogram obtained from HCA (see Fig. 4C). Useful information on PC species exhibiting more relevance in terms of discrimination observed between sample types was inferred from the PCA biplot, reported in Supplemental Fig. S6 (Supplementary materials), and in the heatmap of HCA, shown in Fig. 4C. Indeed, most species contributing to the negative value of PC1, characteristic of Mfn1/2^−/−^ samples (indicated by numbers 7, 8, and 9 in the biplots of Supplemental Fig. S6) were diacylic PCs. Consistently, these compounds generally exhibited the highest autoscaled values, represented by a light to dark orange color, in the HCA heatmap when Mfn1/2^−/−^ samples were considered. As for the WT versus OPA1^−/−^ comparison, the PC biplot in Supplemental Fig. S6 emphasized the incidence of PC O species with sum compositions 34:4, 36:4, 38:7, and 40:8 in OPA1^−/−^ mitochondria (samples 4, 5, and 6 in the biplot). Conversely, PC O species with a generally lower ratio between C=C bonds and carbon atoms of side chains were more specific of WT mitochondria (samples 1, 2, and 3 in Supplemental Fig. S6). This feature, suggesting a better packing of PCs in the mitochondrial membranes’ bilayer, was observed also for species including 38 and 40 carbon atoms, specifically abundant in WT samples 2 and 3, as clearly inferred from the HCA heatmap. Not surprisingly, these samples were grouped in a sub-cluster of the WT samples cluster in the HCA dendrogram (see Fig. 4C).

#### Phosphatidylethanolamines

**Fig. 5:**
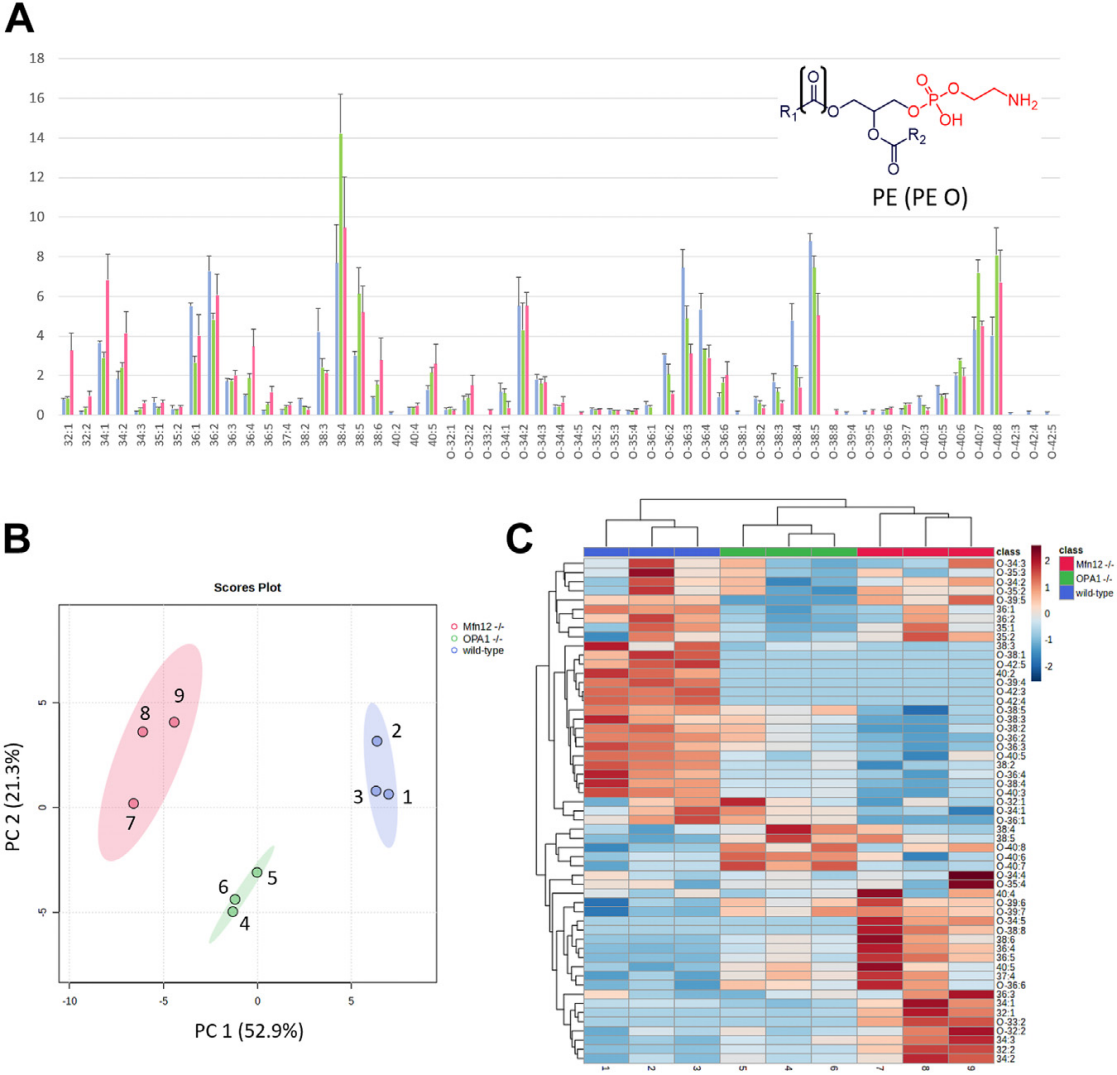
Chemometrics based on the intra-class profile of PEs and PE Os enables a distinction between mitochondria of WT, OPA1^−/−^, and Mfn1/2^−/−^ MEFs. A: Intraclass abundance profile for PE and PE O obtained after the HILIC-ESI(−)-FTMS analysis of mitochondrial lipid extracts referred to WT (light blue columns), OPA1^−/−^ (green columns), and Mfn1/2^−/−^ (red columns) MEFs. Average values for three biological replicates are reported; error bars represent standard deviations. B: Score plot referred to the first two principal components obtained after PCA based on intraclass abundances of PE and PE O, including 95% confidence regions for each type of sample. C: Sample and variable dendrograms with heatmap resulting from the HCA based on the same input data. Autoscaled values were adopted in both cases; Euclidean distances and the average linkage agglomeration algorithm were adopted for HCA. FTMS, Fourier-transform mass spectrometry; HCA, hierarchical cluster analysis; HILIC, hydrophilic interaction liquid chromatography; MEF, mouse embryonic fibroblast; Mfn 1/Mfn 2, mitofusin 1/mitofusin 2; OPA1, optic atrophy 1; PCA, principal components analysis; PE, phosphatidylethanolamine.

As evidenced in Fig. 5A, the distribution of PEs appeared more homogeneous between different sum compositions, 21 of which were referred to diacylic species and 34 to alk(en)yl/acylic ones. Similarly to PCs, diacylic PEs were generally more abundant in Mfn1/2^−/−^ samples. Interestingly, PE O species including 40 carbon atoms on their side chains showed a progressive increase in relative abundance when the number of C=C bonds increased from 3 to 8. Once again, differences between WT and OPA1^−/−^ mitochondria were subtler and data processing by chemometrics was helpful to evaluate them more deeply. Indeed, the PCA score plot obtained from the first two principal components (explaining 74.2% of total variance) showed a nice separation between the three sample types, despite the higher internal variability observed for Mfn1/2^−/−^ samples (see Fig. 5B). The outcome of HCA was consistent, as evidenced by the sample dendrogram reported in Fig. 5C. As inferred from the PCA biplot for PEs (see Supplemental Fig. S6 in the Supplementary material) and the HCA heatmap of Fig. 5C, diacylic PE were generally more relevant in the case of Mfn1/2^−/−^ samples, yet PE O with 32–34 carbon atoms and 1–4 C=C bonds on side chains were also relevant for them, especially in the case of samples 8 and 9. Conversely, sample 7 exhibited a characteristic abundance of some highly unsaturated PE Os (36:6, 34:5, and 38:8), along with PE 36:4, 36:5, 38:6, and 40:5, which likely determined its separation from the sub-cluster of samples 8 and 9 in the HCA dendrogram. As for OPA1^−/−^ samples, the HCA heatmap evidenced the role of other long chain/highly unsaturated PE Os (40:6, 40:7 and 40:8) and of diacylic PC 38:4 and 38:5 as discriminating variables, as confirmed by the PCA biplot for PEs shown in Supplemental Fig. S6. Finally, species more typical of WT samples were PE Os including a generally low number of C=C bonds, compared to the length of side chains, thus marking a remarkable similarity with PCs. The implications of the findings obtained for PCs and PEs in terms of the shape and stability of mitochondrial membranes, and the influence of OPA1 and Mfns depletion on PL biosynthetic pathways will be discussed later.

#### Phosphatidylinositoles

**Fig. 6:**
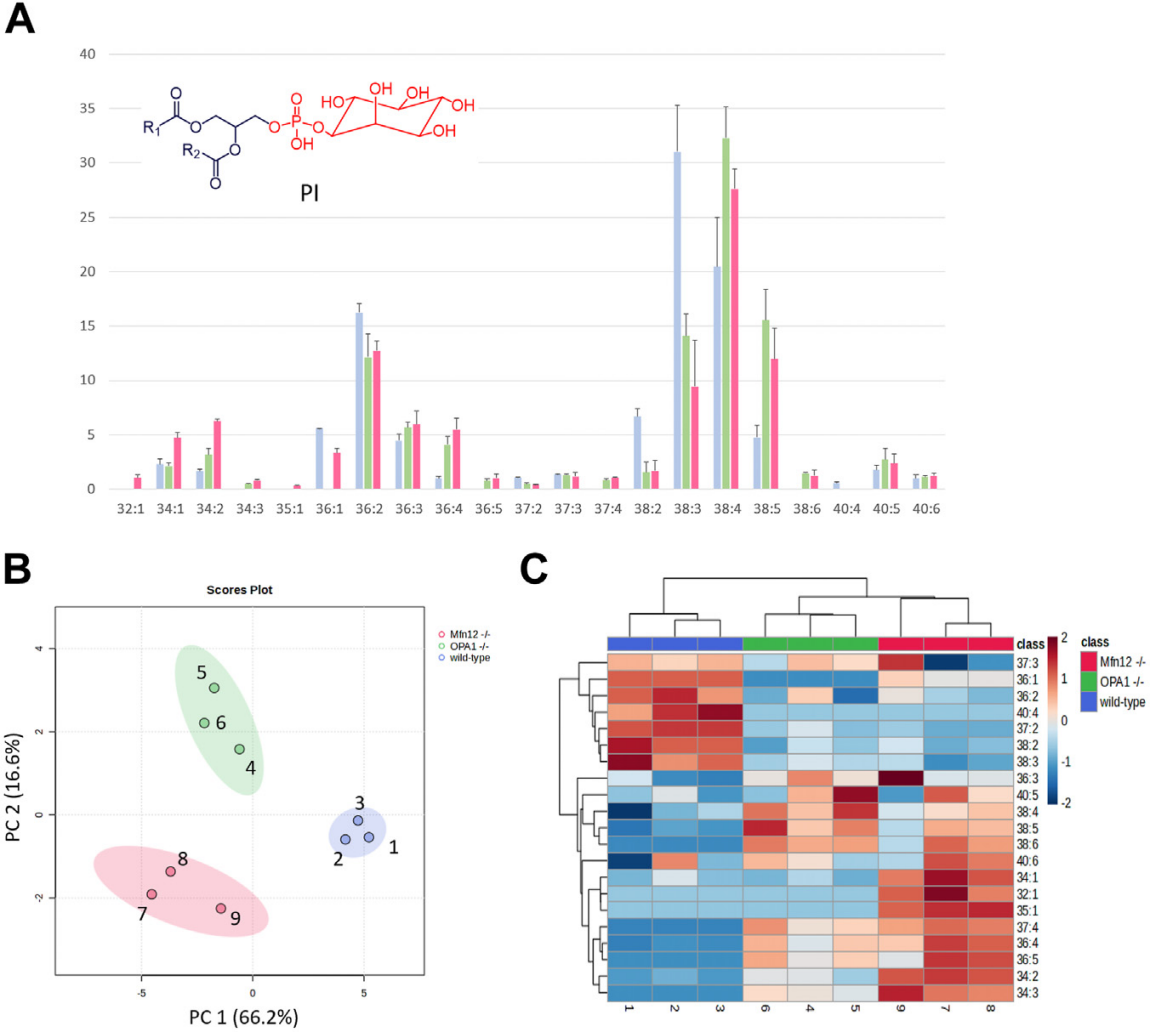
The intraclass profile of PIs enables a distinction between mitochondria of WT, OPA1^−/−^, and Mfn1/2^−/−^ MEFs. A: Intraclass abundance profile for PI obtained after the HILIC-ESI(−)-FTMS analysis of mitochondrial lipid extracts referred to WT (light blue columns), OPA1^−/−^ (green columns), and Mfn1/2^−/−^ (red columns) MEFs. Average values for three biological replicates are reported; error bars represent standard deviations. B: Score plot referred to the first two principal components obtained after PCA based on intraclass abundances of PI, including 95% confidence regions for each type of sample. C: Sample and variable dendrograms with heatmap resulting from the HCA based on the same input data. Autoscaled values were adopted in both cases; Euclidean distances and the average linkage agglomeration algorithm were adopted for HCA. FTMS, Fourier-transform mass spectrometry; HCA, hierarchical cluster analysis; HILIC, hydrophilic interaction liquid chromatography; MEF, mouse embryonic fibroblast; Mfn 1/Mfn 2, mitofusin 1/mitofusin 2; OPA1, optic atrophy 1; PCA, principal components analysis; PI, phosphatidylinositol.

The abundance profiles of PI were simpler than those of PCs and PEs, due to the lower number of sum compositions detected (21 in total, see Fig. 6A). As already mentioned, only diacylic species were found for this class, basically belonging to three major subgroups, corresponding to side chains with 38, 36, and 34 carbon atoms, in order of decreasing abundance. Interestingly, a defined trend in the number of C=C bonds was observed in each of those subgroups, with more unsaturated species being more abundant in Mfn 1/2^−/−^ and OPA1^−/−^ MEF mitochondria. As emphasized in Fig. 6B, C, a clear distinction was observed between the three types of samples both in the PCA score plot for the first two principal components (accounting for 82.8% of total variance) and in the sample dendrogram obtained using HCA. The PCA biplot for PI (see Supplemental Fig. S7 in the Supplementary materials), and also the HCA heatmap (see Fig. 6C) evidenced the low ratio between the numbers of C=C bonds (1, 2, 3, 4) and carbon atoms (36, 37, 38, 39, 40) on acylic side chains occurring for PIs more specific for WT mitochondria (samples 1, 2, and 3). In the case of OPA1^−/−^ mitochondria (samples 4, 5, and 6), prevailing PIs had a similar side chain overall length compared to WT ones (38, 39, 40) but a higher number of C=C bonds (4–6). Finally, more relevant PIs in Mfn1/2^−/−^ mitochondria displayed a wider range of side chain compositions (from 32 to 40), with the number of C=C bonds progressively increasing with that of carbon atoms (from 1–3 for 32–34 atoms up to 6 for species including 38–40 atoms on the acyl chains).

Based on these results, the general composition of PIs seemed to shift from less unsaturated to progressively more unsaturated species in the order: WT < Mfn1/2^−/−^ < OPA1^−/−^, thus confirming the increase in side chain unsaturation observed for PCs and PEs when fusion proteins were absent.

#### Cardiolipins

**Fig. 7:**
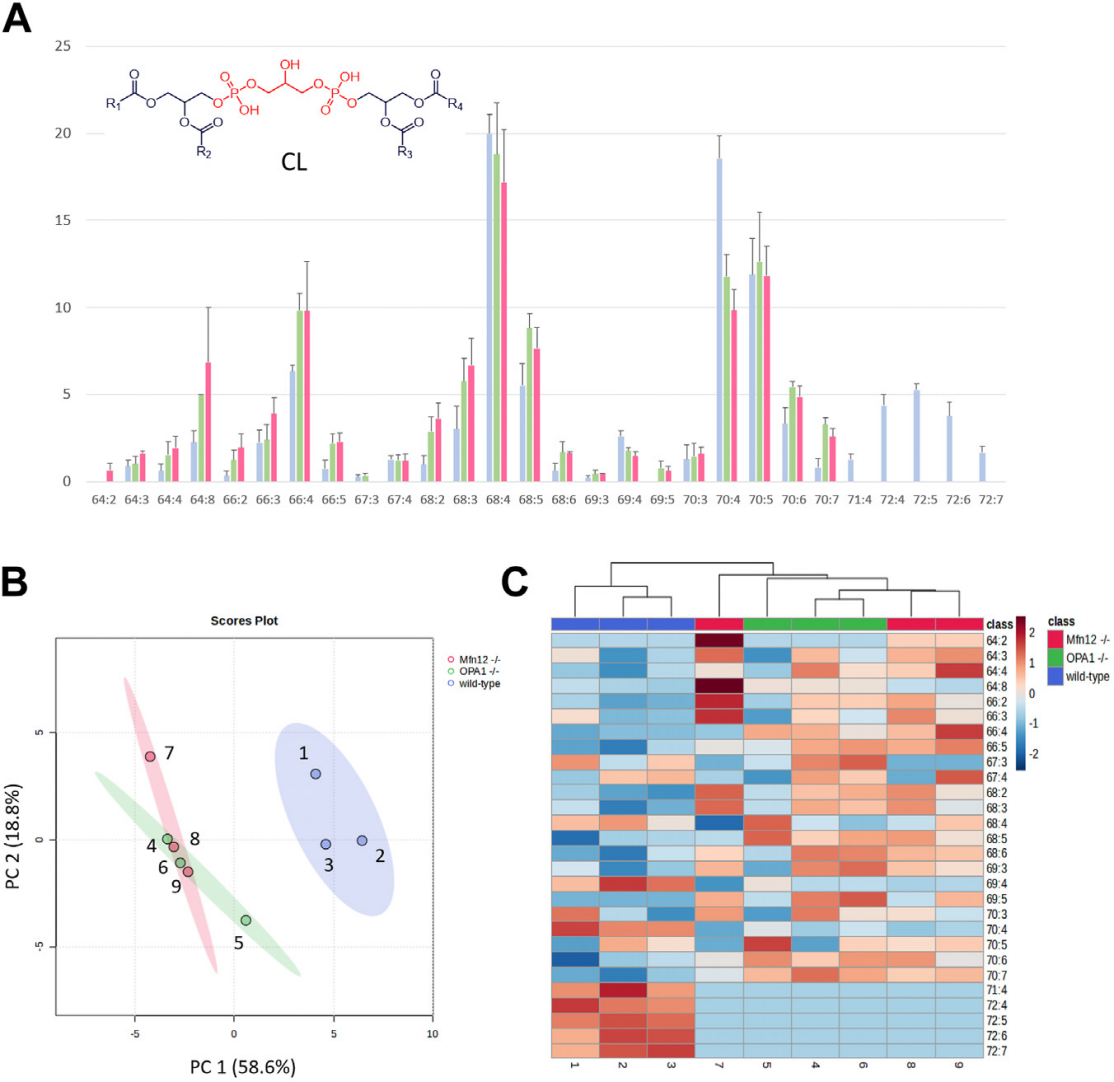
The CL profiles of mitochondria in OPA1^−/−^ and Mfn1/2^−/−^ MEFs do not appear to differ significantly. A: Intraclass abundance profile for CL obtained after the HILIC-ESI(−)-FTMS analysis of mitochondrial lipid extracts referred to WT (blue columns), OPA1^−/−^ (green columns), and Mfn1/2^−/−^ (red columns) MEFs. Average values for three biological replicates are reported; error bars represent standard deviations. B: Score plot referred to the first two principal components obtained after PCA based on intraclass abundances of CL, including 95% confidence regions for each type of sample. C: Sample and variable dendrograms with heatmap resulting from the HCA based on the same input data. Autoscaled values were adopted in both cases; Euclidean distances and the average linkage agglomeration algorithm were adopted for HCA. CL, cardiolipin; FTMS, Fourier-transform mass spectrometry; HCA, hierarchical cluster analysis; HILIC, hydrophilic interaction liquid chromatography; MEF, mouse embryonic fibroblast; Mfn 1/Mfn 2, mitofusin 1/mitofusin 2; OPA1, optic atrophy 1; PCA, principal components analysis.

A characteristic distribution of species was found for major subgroups of the 28 CLs detected in the three types of mitochondria under study, that is, those corresponding to overall side chain lengths of 64, 66, 68, 70, and 72 carbon atoms (see Fig. 7A). Notably, CLs with shorter side chains (*e.g.*, 64, 66, and 68) prevailed in Mfn1/2^−/−^ and OPA1^−/−^ MEF mitochondria, while those including 70 and 72 carbon atoms were either more abundant or exclusively found in WT MEF mitochondria, respectively. This explains why CL 72:x species were crucial for the separate clustering of WT samples in the HCA sample dendrogram (see Fig. 7C) and in the score plot referred to the first two principal components (explaining 74.4% of the total variance), as evidenced by the biplot for CLs reported in Supplemental Fig. S7. Conversely, Mfn1/2^−/−^ and OPA1^−/−^ samples appeared overlapped in the output of both chemometric approaches. Indeed, the 95% confidence regions for these two groups of samples crossed each other in the PCA score plot (see Fig. 7B). Consistently, the corresponding subclusters were mixed in the HCA sample dendrogram (see Fig. 7C), with the Mfn1/2^−/−^ sample 7 being unique, due to the abundance of sum compositions 64:2, 64:3, 64:8, 66:2, and 66:3.

## Discussion

The use of HILIC-ESI-FTMS analysis enabled a very detailed description of the profile of major PL classes in mitochondria of WT, OPA1^−/−^, and Mfn1/2^−/−^ MEFs, providing an unprecedented level of detail for PCs, PEs, and PIs and confirming the profile previously reported for CLs in the case of MEF depleted of OPA1 (25). Only a few species in each class displayed statistically significant differences across the three types of samples. Nonetheless, the main goal of the present study was not finding specific discriminating lipid markers, but rather retrieving a global picture of the PL profiles and evaluating if and how the absence of fusion proteins might affect them.

Processing by unsupervised chemometrics was able to emphasize the subtle differences occurring between the three sample types, revealing a general trend: mitochondria lacking fusion proteins had a higher proportion of PLs with more double bonds in their acyl chains. Additionally, a progressive reduction of the incidence of alk(en)ylic/acylic species with respect to their acylic counterparts in PEs and, at a lower extent, in PCs was observed when comparing WT mitochondria with OPA1^−/−^ and Mfn1/2^−/−^ ones.

A comparison of our results with those reported in the literature, starting from concentration ratios between major PL classes, could be done only considering data referred to whole cell lipid extracts obtained from WT and/or OPA1^−/−^ MEFs (25, 26). Conversely, no data were found in the literature on the PL profiles of Mfn1/2^−/−^ MEF cells or of their mitochondria, thus the present study provided, to the best of our knowledge, the first information on this type of samples.

Both the PE/PC ratio (0.22 vs. 0.21) and the PI/PC ratio (0.11 vs. 0.12) inferred in Ref. (26) from the analysis of whole cell lipid extracts of WT and OPA1^−/−^ MEFs appeared unaltered by the lack of OPA1, consistently with the findings of the present study. No comparison could be done for the CL/PC ratio, since the authors were unable to detect CLs, likely because the contribution of these mitochondria-specific PL to the whole cell phospholipidome was limited. Cretin *et al.* (25) studied WT and OPA1^Crispr^ MEFs, which can be considered comparable to our WT and OPA1^−/−^ MEFs, respectively, although OPA1^Crispr^ MEFs were expected to be not totally deprived of OPA1. The PL class ratios inferred from their data for the two cell lines (0.20 vs. 0.25 for PE/PC and 0.13 vs. 0.15 for PI/PC, see Fig. 5E in Ref. (25) were similar to those reported in Ref. (26) and, once again, did not show a significant change related to the lack of OPA1. Interestingly, data on CLs could be also obtained in the same study; in particular, the CL/PC ratios for WT + NT siRNA and OPA1^Crispr^ + NT siRNA MEFs could be calculated as 0.070 and 0.065, respectively (25). Even if these values are much lower than those found in our case, since CLs were minor PLs in the total cell lipid extracts, they indicated the depletion of OPA1 to be unable to alter the CL/PC ratio. The comparisons now discussed strengthen the evidence that major PL classes concentrations remain stable in OPA1-deficient cells, both at the whole cell and at the mitochondrial level. Notably, we observed a similar stability even in the absence of Mfn 1 and Mfn 2, a condition not previously explored.

Regarding intraclass concentration ratios involving alk(en)ylic/acylic PC or PEs, estimates of their values from data reported by Bocca *et al.* (26), referred to whole MEFs, indicated a significant increase of the PC O/PC ratio when OPA1 was lacking, whereas the PE O/PE ratio was substantially unaltered. This discrepancy with our data, specific for mitochondrial PCs and PEs, suggests that the lack of OPA1 and Mfn1/2 might affect the transfer of alk(en)yl/acyl PC and PE species to mitochondria (vide infra). CLs were the only PLs for which a direct comparison of intraclass abundance profiles could be done between our data and those obtained in previous studies, considering the results reported for whole cell extracts of WT and OPA1^Crispr^ MEFs (25). Major sum compositions found for CLs in the present study, that is, 68:4, 70:4, and 70:5, were also the prevailing ones reported for these samples, thus indicating a good consistency between similar cell lines.

As a general consideration, our data suggest the occurrence of limited changes in the abundance profile of major PL classes (PCs, PEs, PIs, and CLs) in MEF mitochondria lacking OPA1 or Mfn1 and Mfn 2. To find a correlation between this finding and the ultrastructure of MEF mitochondria, we reviewed the literature in which the latter was studied using transmission electron microscopy. Over a decade of research (25, 42, 43, 44, 45, 46, 47, 48) has shown that the lack/depletion of OPA1 leads to significant alterations in mitochondrial *cristae*, with a dramatic reduction of their number (down to 2–4 per mitochondrion), and to a more rounded shape compared to WT cell mitochondria. This feature was also quantitatively assessed in a recent study relying on a deep learning–based image analysis platform (48), showing that the maximum length of mitochondria in OPA1 knockdowns of mouse fibroblasts was significantly reduced compared to that observed in WT cells, whereas minimum lengths remained substantially unaltered (see Table 1 in Ref. (48). Changes in mitochondrial shape and number of *cristae* similar to those found for OPA1 KO or knockdown were observed for mitochondria of Mfn1/2^−/−^ MEFs (49) and those located in the skeletal muscles of Mfn double mutant mice (50).

Notably, PCs and PIs are known to promote the stability of the membrane bilayer, due to their cylindrical shape (51). Conversely, PEs and CLs are conical-shaped and known to induce a curvature stress on membranes, thus they are classified as nonbilayer forming PLs (51). Consequently, *cristae*, that are characterized by a high curvature, exhibit the highest incidence of PEs and CLs within the IMM (52). Surprisingly, despite the *cristae* structure is remarkably disrupted in mitochondria lacking OPA1 or Mfns, the incidence of PEs and CLs with respect to PCs inferred from our HILIC-ESI-FTMS analyses was not significantly lowered in them. Indeed, only a slight decrease was observed for the CL/PC ratio in Mfn1/2^−/−^ mitochondria (see Fig. 3). A possible explanation for this finding might be related to the generally increased curvature of both the OMM and the IMM in OPA1^−/−^ and Mfn1/2^−/−^ MEF mitochondria (42, 43, 44, 45, 49). In fact, PEs and CLs originally located in *cristae* membranes might contribute to the increased incidence of curved domains in the *cristae*-depleted IMM of altered mitochondria; PEs might contribute to the increased curvature of the OMM as well.

The decrease in the incidence of PE Os with respect to PEs in both OPA1^−/−^ and Mfn1/2^−/−^ mitochondria and of PC Os versus PCs in the latter (see Fig. 3) requires a more complex explanation. As evidenced in a recent monography (see Ref. (53) and references cited therein), PLs including an ether-linked side chain, especially those including a vinyl-ether bond, can form more tightly packed membranes compared to their diacylic counterparts. Therefore, the decrease in the incidence of alk(en)yl/acyl PC and PE species related to the lack of OPA1 or Mfn 1 and 2, might further destabilize mitochondrial membranes and be consistent with the significant increase in their curvature.

Another noteworthy finding with potential implications for membrane morphology is the general increase in the unsaturation degree of PLs observed when comparing OPA1^−/−^ and Mfn1/2^−/−^ with WT mitochondria. A less tight packing of PLs in membrane bilayers is expected when their side chains include several C=C bonds, since these are usually characterized by a *cis* geometry, implying the presence of kinks in the chains. Consequently, increased unsaturation is expected to make PL-based membranes more fluid (54). Based on our findings, this could be the case also of OPA1^−/−^ and Mfn1/2^−/−^ MEF mitochondrial membranes.

While PL profiles emerged from the present study were consistent with the existing data on mitochondrial ultrastructure, providing a biosynthetic explanation for the reshaping of PL profiles occurring upon depletion of OPA1 or Mfn1 and 2 is more challenging. Nonetheless, a recent study by Renne *et al.* (55), exploiting pulsed labeling with stable isotope-labeled serine and subsequent analysis of isotopically labeled PSs and PEs by shotgun lipidomics in specific mutants of yeast strains, offers some clues. In particular, mitochondria were shown to preferentially import diunsaturated PSs, subsequently converted to PEs by the mitochondrial PS decarboxylase Psd1p (55). On the other hand, the accumulation of saturated acyl chains in yeast mitochondrial PEs was emphasized by the inactivation of protein complexes known to tether mitochondria to the ER, namely, the ER-mitochondrial encounter and the vacuole and mitochondria patch) structures (55). Although a direct investigation on the expression of these enzymes or protein complexes goes beyond the goals of the present work, a combination of these findings with the results of mitochondrial PL profiling suggest that also in the case of MEFs the lack of OPA1 or Mfns might interfere with the lipid transport from the ER to mitochondria, leading to the accumulation of PL with higher unsaturation levels, in particular PEs and PCs, the latter being strictly related to PEs in terms of biosynthesis (14). Moreover, since PCs and PEs are a source of acyl chains for CLs during their maturation process, that is, the conversion leading to an increase in the unsaturation level of CLs (52), a general increase in the number of C=C bonds of PCs and/or PEs side chains might be reflected in an increasing unsaturation level also for CLs.

Concerning alk(en)ylic/acylic PEs and PCs, it is worth noting that their synthesis starts inside peroxisomes, where the reduction of the C=O group of the acyl moiety of an acyl-CoA molecule is catalyzed by fatty acyl-CoA reductase 1, leading to the corresponding fatty alcohol (56). In a further peroxisomal step, this alcohol is incorporated into an alkylglycerone phosphate molecule, subsequently converted to 1-O-alkyl-sn-glycero-3-phosphate. The final steps in the biosynthesis of PE Os or PC Os from the latter occur in the ER and, according to the most recent update of the biosynthetic pathway (see Ref. (56) and references cited therein), distinct routes are followed for alkyl/acyl (plasmanyl) and alk-1-enyl/acyl (plasmenyl) PEs and PCs. Although, as mentioned before, the distinction between alkyl/acyl and alk-1-enyl/acyl PE and PC in MEF mitochondria was beyond the goals of the present investigation, HILIC-ESI-FTMS analyses clearly indicated a decreasing incidence of alkylic/acylic PEs and PCs in the respective classes when OPA1 and Mfn 1 and 2 were absent. This finding suggests an impaired transport of PE Os and PC Os from the ER to mitochondria as a result of the lack of fusion proteins. However, further investigations are required to evaluate if the latter might even determine a disruption in some of the ER-located pathways leading to those types of PEs and PCs.

The results of the present study can be reasonably interpreted with the occurrence of an altered transport of major PLs, including alk(en)ylic/acylic ones, from the ER to mitochondria when fusion proteins are absent, with remarkable consequences on the mitochondrial morphology and function. This conclusion aligns with studies indicating Mfn 2 as a structural tether between ER and mitochondria (see Ref. (54) and references cited therein). In particular, alternative splicing of the human Mfn2 gene has been recently proposed to result in two additional transcripts coding for as many proteins restricted at the ER, one of which (ER mitofusin 2 tether) might represent the ER partner of mitochondrial Mfn in tethering (57). Further research is needed to explain how the lack of fusion proteins, including OPA1, can alter the normal PL transport to mitochondria. Moreover, it needs to be investigated how the changes observed in the major PL profiles impact on other IMM-linked features associated with absence of the fusion proteins. Specifically, these include the assembly of OXPHOS supercomplexes and possibly the maintenance of mtDNA, assuming that mtDNA nucleoids are tightly associated to the IMM. Both are key aspects of the fusion-dependent cascade of mitochondrial dysfunctions.

## Data Availability

Data reported in the present study can be shared upon request to Prof Ilario Losito, University of Bari “Aldo Moro,” Department of Chemistry, e-mail: ilario.losito@uniba.it.

## Supplemental data

This article contains supplemental data.

## Conflict of interest

The authors declare that they have no conflicts of interest with the contents of this article.
