# Supplementary material for: Lipidomics reveals the reshaping of the mitochondrial phospholipid profile in cells lacking OPA1 and mitofusins
Source: J Lipid Res. 2024 May 18;65(6):100563. doi: 10.1016/j.jlr.2024.100563 (PMC11225846; doi:10.1016/j.jlr.2024.100563)
Supplement: Supplemental Data [file mmc1.docx]

**Supplementary Materials**

**Lipidomics reveals the reshaping of the mitochondrial phospholipid profile in cells lacking OPA1 and Mitofusins**

Andrea Castellaneta^1^, Ilario Losito^1,2,*^, Vito Porcelli^3^, Serena Barile^3^, Alessandra Maresca^4^, Valentina Del Dotto^5^, Valentina Losacco^1^, Ludovica Sofia Guadalupi^1^, Cosima Damiana Calvano^1,2^, David C. Chan^6^, Valerio Carelli^4,5^, Luigi Palmieri^3,7^, Tommaso R.I. Cataldi^1,2^

*^1^Dipartimento di Chimica, ^2^Centro Interdipartimentale SMART and ^3^Dipartimento di Bioscienze, Biotecnologie e Ambiente - Università degli Studi di Bari Aldo Moro, via Orabona 4, 70126 Bari, Italy;*

*^4^IRCCS Istituto delle Scienze Neurologiche di Bologna, Programma di Neurogenetica, via Altura 3, 40139 Bologna, Italy;*

*^5^Dipartimento di Scienze Biomediche e Neuromotorie, Università degli Studi di Bologna, via Altura 3, 40139 Bologna, Italy;*

*^6^Division of Biology and Biological Engineering, California Institute of Technology, 1200 East California Boulevard, 91125 Pasadena, CA, USA.*

*^7^CNR-Istituto di Biomembrane, Bioenergetica e Biotecnologie Molecolari, Via Giovanni Amendola, 122/O, 70126 Bari, Italy*

**
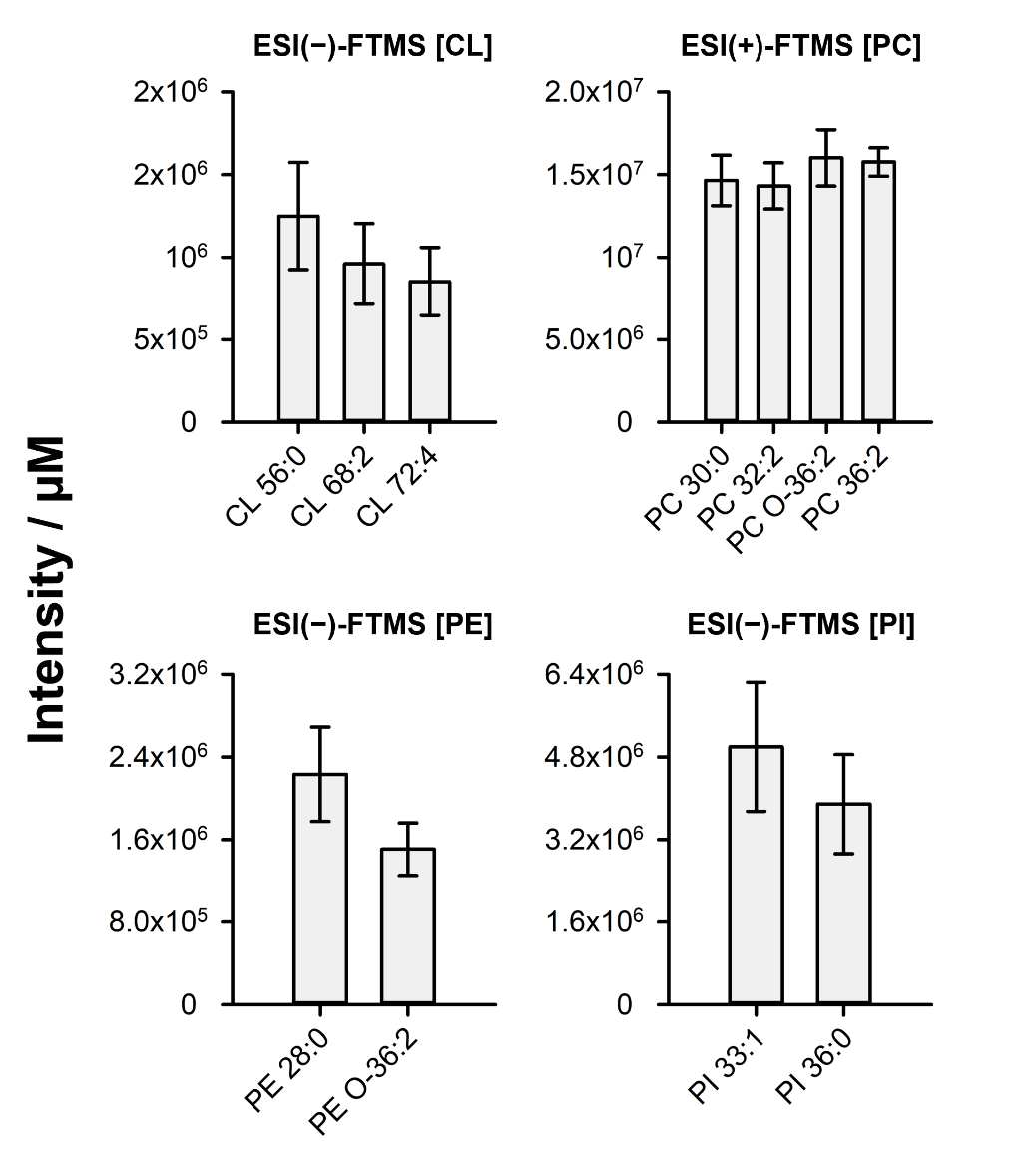
Figure S1**. Graphical representation of the confidence intervals (α=0.05) obtained for the slopes of the calibration lines referred to several PL species in a concentration range from 0.01 to 10 µM, after the HILIC-ESI-FTMS analysis of their mixtures under the same conditions adopted for mitochondrial lipid extracts. Once subjected to Type-I correction, the intensities of the MS signals in the FTMS spectra averaged under the HILIC peaks pertaining to each lipid class were exploited as the analytical response. CL 14:0/14:0/14:0/14:0 (CL 56:0), CL 16:0/18:1/16:0/18:1 (CL 68:2), and CL 18:1/18:1/18:1/18:1 (CL 72:4) were considered as representative for CLs. PC 14:0/16:0 (PC 30:0), PC 16:1/16:1 (PC 32:2), PC P-18:0/18:1 (PC O-36:2), and PC 18:1/18:1 (PC 36:2) were considered as representative for PCs. PE 14:0/14:0 (PE 28:0) and PE P-18:0/18:1 (PE O-36:2) were considered as representative for PEs. PI 15:0/18:1 (PI 33:1) and PI 18:0/18:0 (PI 36:0) were considered as representative for PIs.


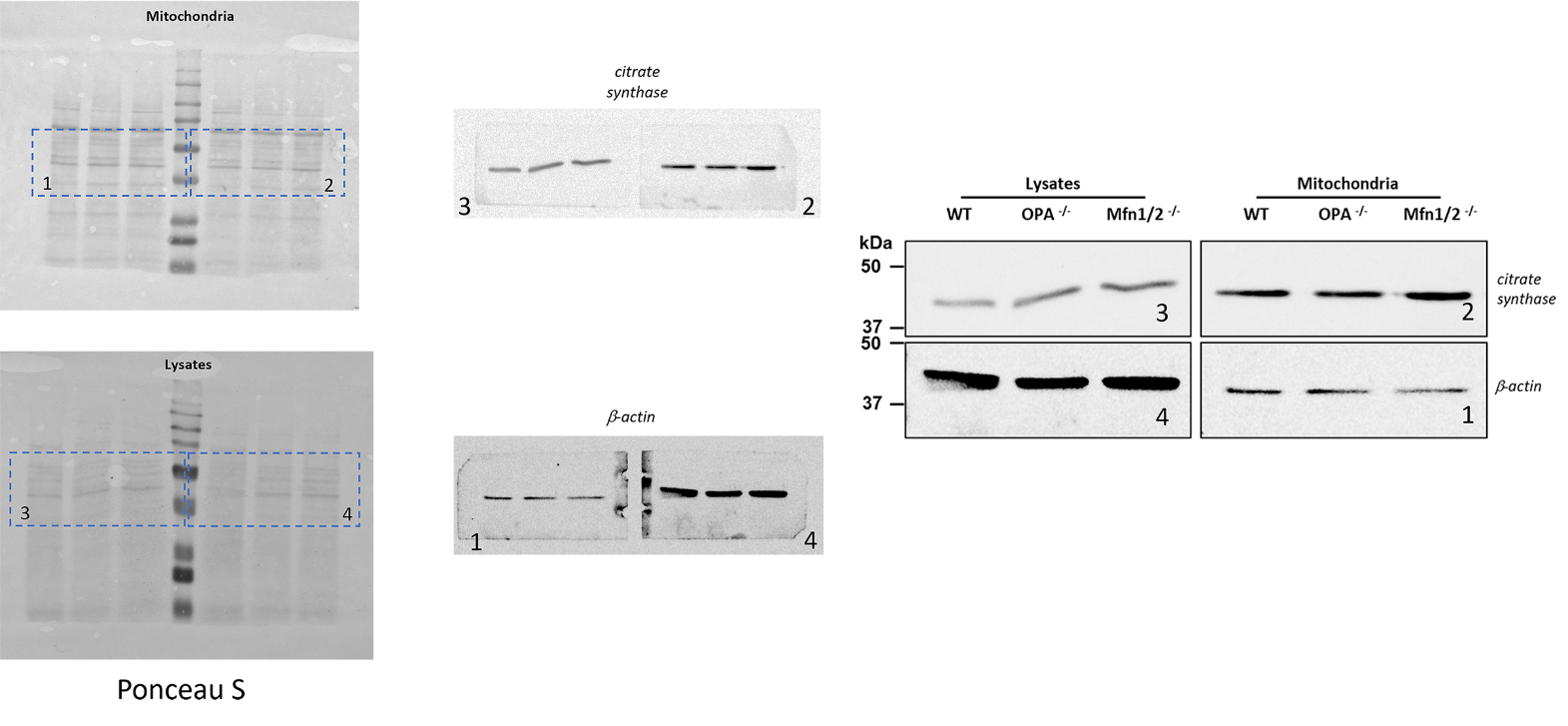


**Figure S2**. Details on the procedure adopted to obtain cropped gel images reported in panel A of Figure 1. The gels, loaded with mitochondrial fractions and cellular lysates separately, were transferred onto nitrocellulose membranes and stained with Ponceau S (A and B respectively). Afterwards, the membranes were cut where indicated. Sections 3 and 2 were incubated with an antibody against citrate synthase whereas sections 1 and 4 with an antibody against β-actin, as described in the main manuscript.

**
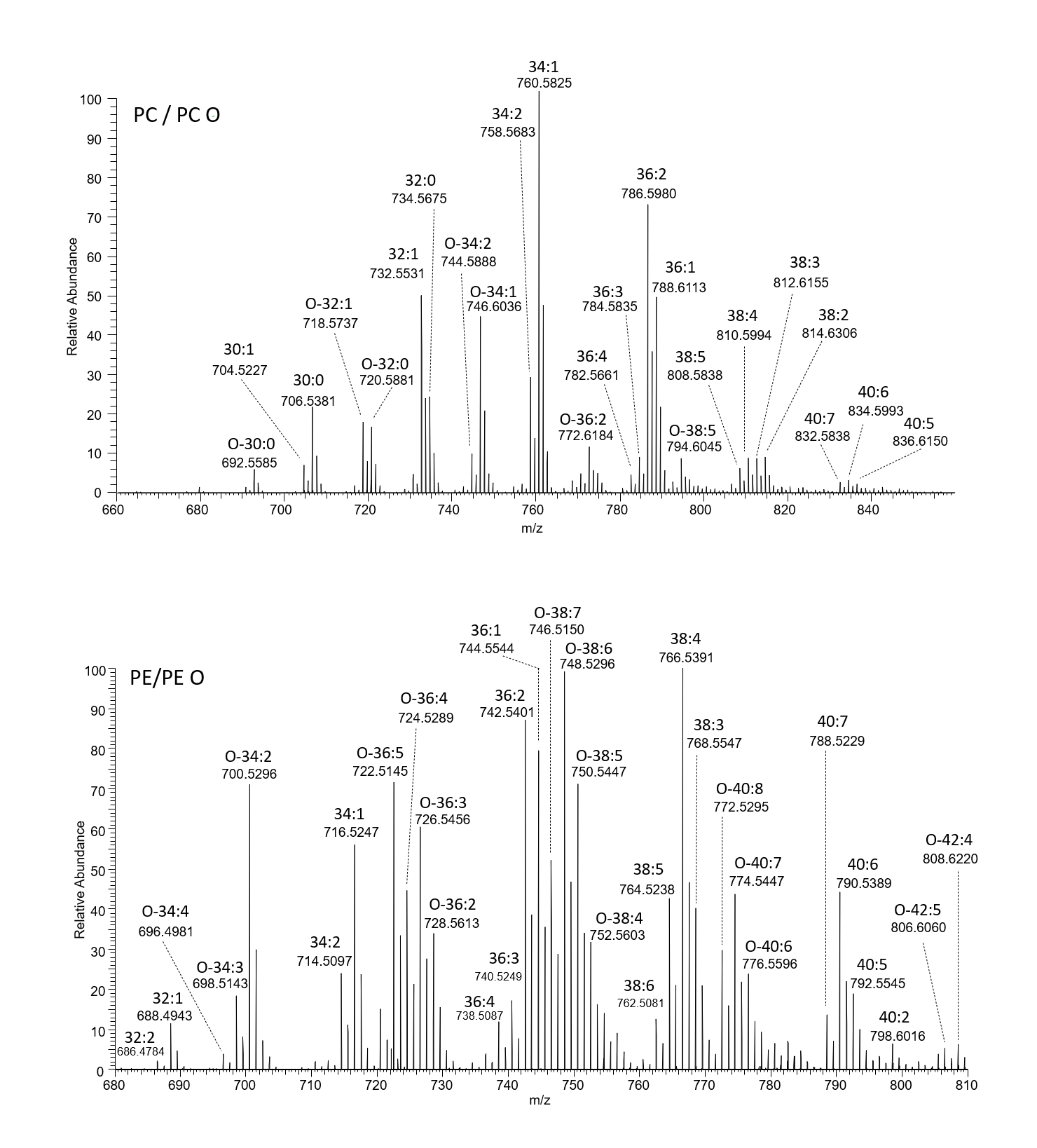
**

Figure S3. FTMS spectra averaged under chromatographic bands referred to PC and PC O (upper panel), and PE and PE O (lower panel), obtained by HILIC-ESI-FTMS analysis, in positive and negative polarity, respectively. The sample was a lipid extract of mitochondria isolated from wild-type MEFs. For the sake of clarity, peak signals referred to more abundant monoisotopic ions were labelled with the sum composition of the corresponding PL. In the case of alk(en)yl/acyl PL the sum composition label is reported as O-C:D, with C and D representing the total number of carbon atoms and C=C bonds of the side chains.


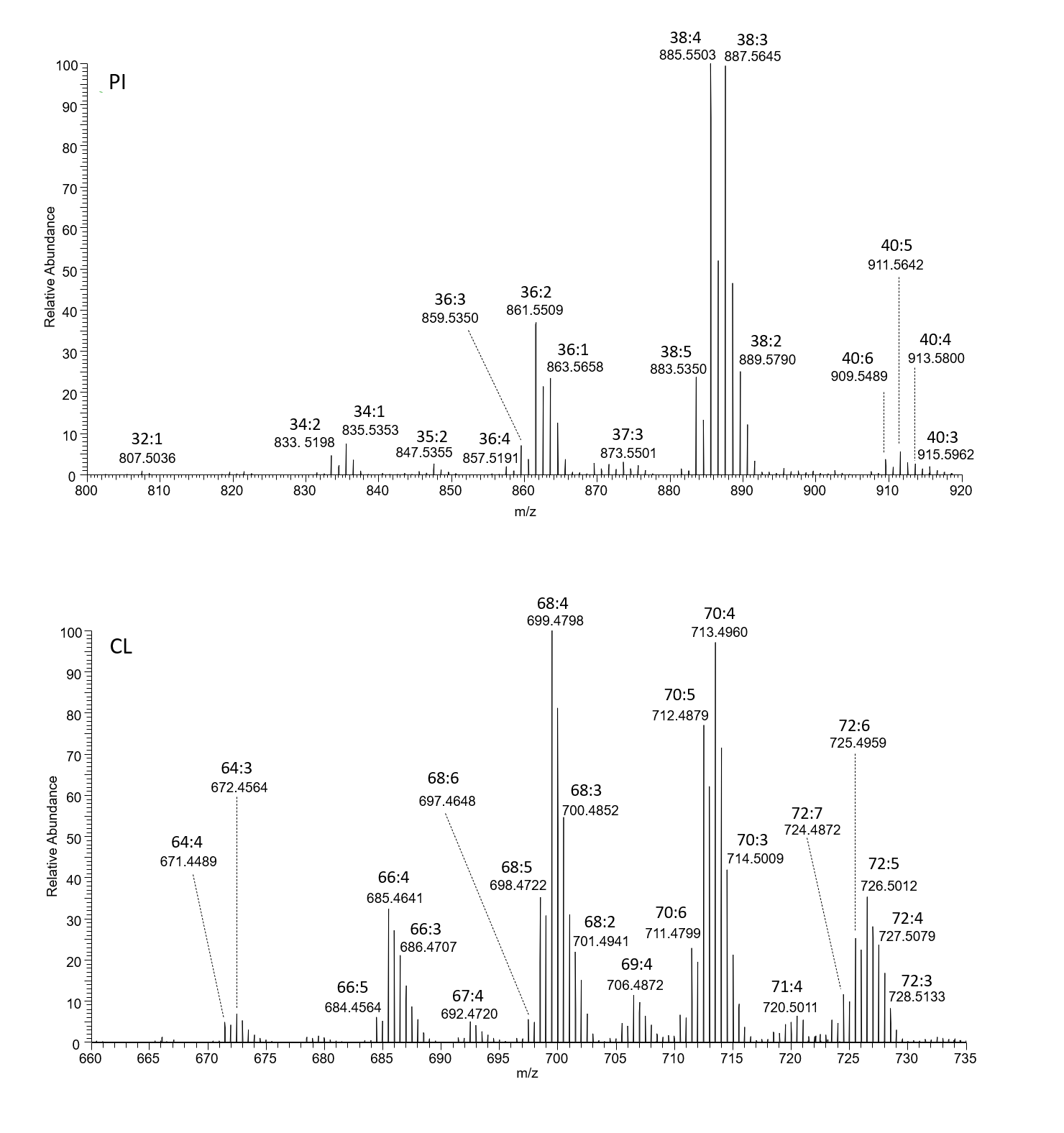


Figure S4. FTMS spectra averaged under chromatographic bands referred to PIs (upper panels) and CLs (lower panel) obtained by HILIC-ESI-FTMS analysis in negative polarity. The sample was a lipid extract of mitochondria isolated from wild-type MEFs. The *m/z* interval shown for CL is referred to the corresponding [M-2H]^2-^ ions. For the sake of clarity, peak signals referred to more abundant monoisotopic ions were labelled with the sum composition of the corresponding PLs.


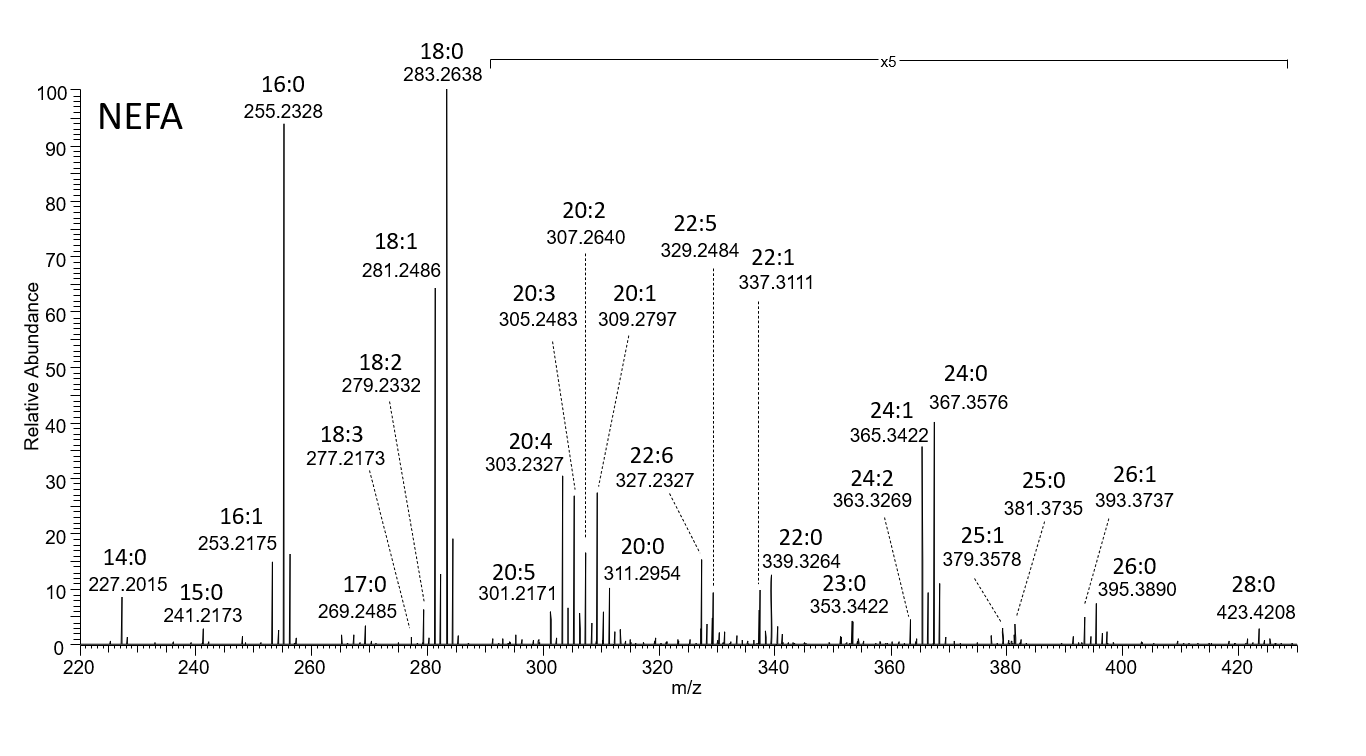


**Figure S5.** Typical FTMS spectrum obtained for non-esterified fatty acids (NEFA) after spectral averaging under the corresponding band detected in the HILIC-ESI(-)-FTMS chromatogram referred to the mitochondrial lipid extract of wild-type MEF. A 5-fold vertical magnification was performed for the *m/z* interval 290-430, to facilitate the observation of peak signals referred to longer side chain NEFA (20 to 28 carbon atoms).


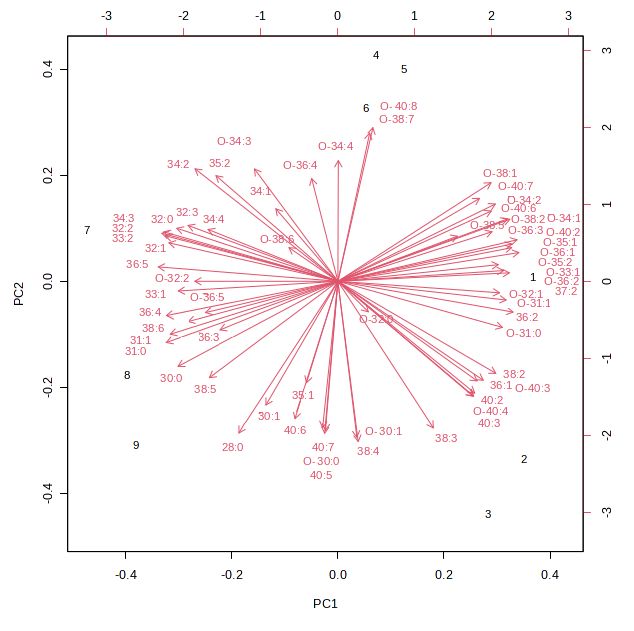


PC


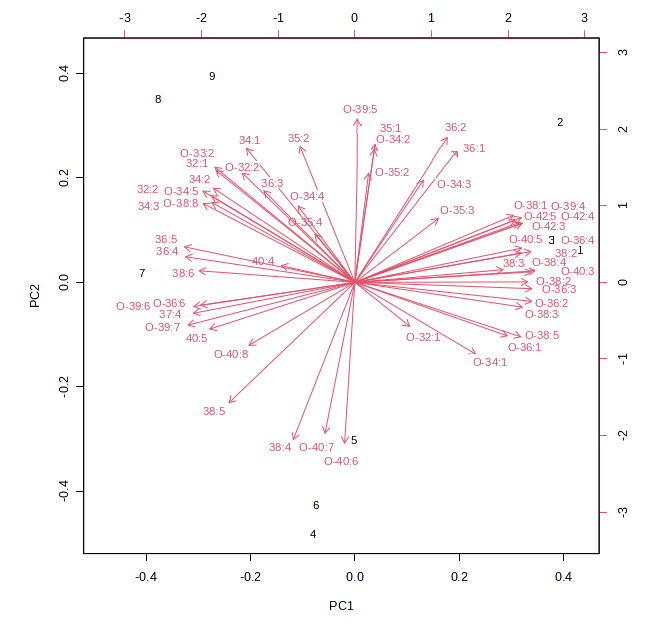


PE

**Figure S6**. Biplots obtained for the first two principal components after the PCA based on intra-class relative abundances of PC and PE species, estimated after the HILIC-ESI-FTMS analysis of mitochondrial lipid extracts for wild-type (indicated as samples #1, 2, and 3 in the figure), OPA1^-/-^ (samples #4, 5 and 6) and Mfn1/2^-/-^ (samples #7, 8 and 9) MEF.


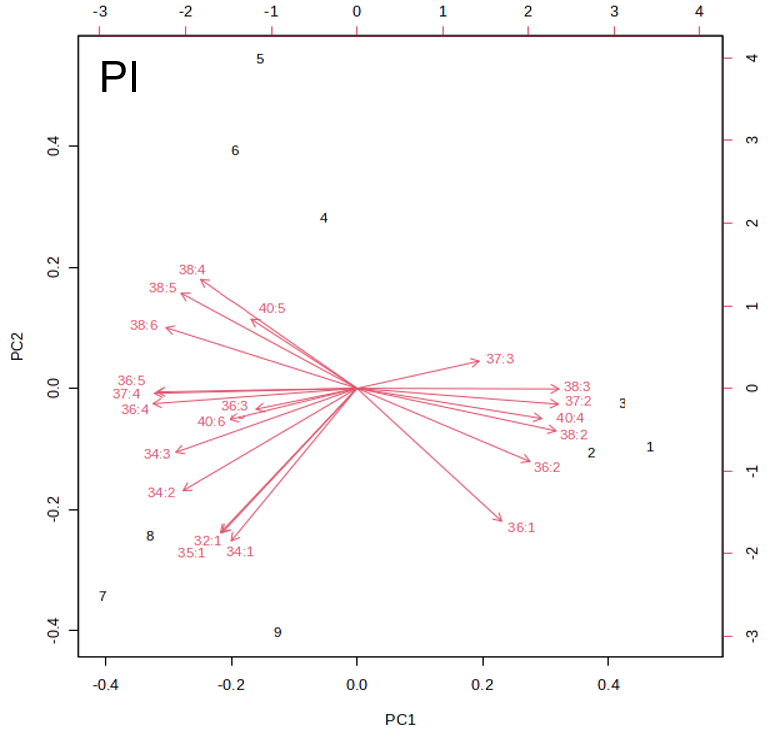


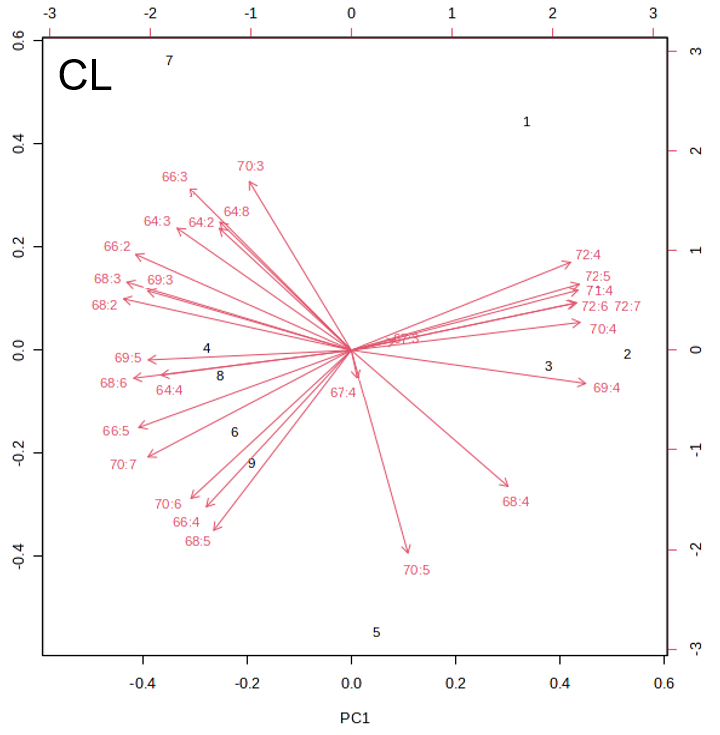


**Figure S7**. Biplots obtained for the first two principal components after the PCA based on intra-class relative abundances of PI and CL species, estimated after the HILIC-ESI-FTMS analysis of mitochondrial lipid extracts for wild-type (indicated as samples #1, 2, and 3 in the figure), OPA1^-/-^ (samples #4, 5 and 6) and Mfn1/2^-/-^ (samples #7, 8 and 9) MEF.
